# Online eLearning for undergraduates in health professions: A systematic review of the impact on knowledge, skills, attitudes and satisfaction

**DOI:** 10.7189/jogh.04.010406

**Published:** 2014-06

**Authors:** Pradeep Paul George, Nikos Papachristou, José Marcano Belisario, Wei Wang, Petra A Wark, Ziva Cotic, Kristine Rasmussen, René Sluiter, Eva Riboli–Sasco, Lorainne Tudor Car, Eve Marie Musulanov, Joseph Antonio Molina, Bee Hoon Heng, Yanfeng Zhang, Erica Lynette Wheeler, Najeeb Al Shorbaji, Azeem Majeed, Josip Car

**Affiliations:** 1 Health Services and Outcomes Research, National Healthcare Group, Singapore; 2 Global eHealth Unit, Department of Primary Care and Public Health, School of Public Health, Imperial College London, London, UK; 3 Department of Health Evidence, Radboud University Nijmegen, Nijmegen, The Netherlands; 4 Department of Integrated Early Childhood Development, Capital Institute of Pediatrics, Chaoyang District Beijing, P.R. China; 5 Department of Primary Care and Public Health, School of Public Health, Imperial College London, London, UK; 6 Knowledge, Ethics and Research, World Health Organization, Geneva, Switzerland; 7 Health Services and Outcomes Research Programme, Lee Kong Chian School of Medicine, Imperial College & Nanyang Technological University, Singapore; * Joint first authors

## Abstract

**Background:**

Health systems worldwide are facing shortages in health professional workforce. Several studies have demonstrated the direct correlation between the availability of health workers, coverage of health services, and population health outcomes. To address this shortage, online eLearning is increasingly being adopted in health professionals’ education. To inform policy–making, in online eLearning, we need to determine its effectiveness.

**Methods:**

We performed a systematic review of the effectiveness of online eLearning through a comprehensive search of the major databases for randomised controlled trials that compared online eLearning to traditional learning or alternative learning methods. The search period was from January 2000 to August 2013. We included articles which primarily focused on students' knowledge, skills, satisfaction and attitudes toward eLearning and cost-effectiveness and adverse effects as secondary outcomes. Two reviewers independently extracted data from the included studies. Due to significant heterogeneity among the included studies, we presented our results as a narrative synthesis.

**Findings:**

Fifty–nine studies, including 6750 students enrolled in medicine, dentistry, nursing, physical therapy and pharmacy studies, met the inclusion criteria. Twelve of the 50 studies testing knowledge gains found significantly higher gains in the online eLearning intervention groups compared to traditional learning, whereas 27 did not detect significant differences or found mixed results. Eleven studies did not test for differences. Six studies detected significantly higher skill gains in the online eLearning intervention groups, whilst 3 other studies testing skill gains did not detect differences between groups and 1 study showed mixed results. Twelve studies tested students' attitudes, of which 8 studies showed no differences in attitudes or preferences for online eLearning. Students' satisfaction was measured in 29 studies, 4 studies showed higher satisfaction for online eLearning and 20 studies showed no difference in satisfaction between online eLearning and traditional learning. Risk of bias was high for several of the included studies.

**Conclusion:**

The current evidence base suggests that online eLearning is equivalent, possibly superior to traditional learning. These findings present a potential incentive for policy makers to cautiously encourage its adoption, while respecting the heterogeneity among the studies.

Health care workers are central to any health system; their activities are aimed at enhancing the health of the community, nation or region in general. However, these professionals are distributed unevenly across the globe; countries with lower relative need have the highest numbers of health workers, while those with the greatest burden of disease have a much smaller health workforce. The health worker crisis is at its worst in sub–Saharan Africa and Asia. Incidentally, countries in this region are facing a double burden of both infectious and non–communicable diseases [1], and they lack the funds, technology, infrastructure and trained health workers needed to provide basic health care service [2]. At this juncture; the WHO estimates a shortage of 7.2 million doctors, nurses, midwives and other health care professionals worldwide [3]. Addressing this shortfall in health care professionals through training requires a substantial investment.

Meanwhile, the Internet and the development of information technologies have revitalized the exchange of information and training worldwide. Consequently, eLearning is used increasingly in medical and health professional education, to tackle the global shortage of health workers. eLearning is “an approach to teaching and learning, representing all or part of the educational model applied, that is based on the use of electronic media and devices as tools for improving access to training, communication and interaction and that facilitates the adoption of new ways of understanding and developing learning” [4]. It does not only differ from traditional learning (ie, face–to–face learning that takes place in a classroom environment) in the medium by which learning is delivered [5], but also affects the teaching and learning approaches used. eLearning can take the form of a full eLearning approach, which is entirely driven by technology, or be a mix of the traditional and fully computer–based methodologies (blended learning). Blended learning might be more suitable for health care training because of the need to combine hands–on skills–based training at a practical level as well as self–directed learning [6–10].

Lately, eLearning has started to make way into the developing countries and is believed to have huge potential for governments struggling to meet a growing demand for education while facing an escalating shortage of teachers [11]. However, in both the developing and non–developing countries, network connectivity and bandwidth availability are the key obstacles to the effective delivery of online eLearning [5,12]. Despite this, eLearning presents many opportunities for universities, including the reduction of the delivery costs [13], increased scalability [14], improved access and availability by removing geographical and temporal barriers and allowing access to relevant experts and novel curricula [15].

Online eLearning represents a further evolution of computer–assisted or computer based or offline eLearning and is an important tool in medical training and may transform the way medicine is taught [16]. In the recent years, nearly all medical schools in the USA and Canada employ online course materials [17]. eLearning could be full or blended, full eLearning can be distributed geographically and/or temporally, and communication between student and teacher is handled electronically. This manuscript focuses on online eLearning; systematic review of offline eLearning is published in a parallel article [18].

Online eLearning approaches varied widely in configuration (tutorial, asynchronous discussion, live conferencing, etc.), instructional methods (eg, practice exercises, cognitive interactivity) and presentation [17]. The majority of reviews of effectiveness of online eLearning included observational studies with several methodological deficiencies [16,17,19–22]. This systematic review aims to evaluate the effectiveness of online eLearning from randomised controlled trials conducted among undergraduate health professionals, specifically looking at its impact on students’ knowledge, skills, attitudes and satisfaction.

## METHODS

We conducted a systematic review following the Cochrane methodology [23].

### Search methods for identification of studies

**Electronic searches.** We limited our electronic searches to records published on or after the year 2000 in order to highlight recent developments. We developed a search strategy for MEDLINE (OvidSP) using a combination of keywords and MeSH terms that captured the types of intervention and the types of participants under evaluation in this systematic review (**Table 1**). We adapted the search strategy for use in EMBASE (OvidSP), PsycINFO (Ovid SP), Cochrane Central Register of Controlled Trials (CENTRAL), Web of Science, and Educational Resources Information Center (ERIC) (ProQuest). Where available, we used validated methodological filters to limit our searches to Randomised Controlled Trials (RCTs) and cluster RCTs (cRCTs). We ran the searches in August 2013.

**Table 1:** Search strategy for use in MEDLINE (Ovid SP)*

1.	exp Education, Distance/
2.	educat$.mp.
3.	learn$.mp.
4.	train$.mp.
5.	instruct$.mp.
6.	2 or 3 or 4 or 5
7.	“computer assisted”.mp.
8.	Internet.mp
9.	distance.mp.
10.	web.mp.
11.	online.mp.
12.	virtual.mp.
13.	“mobile phone”.mp.
14.	“cell$ phone”.mp.
15.	smartphone
16.	smart–phone
17.	7 or 8 or 9 or 10 or 11 or 12 or 13 or 14 or 15 or 16
18.	6 adj3 17
19.	exp Computer–Assisted Instruction/
20.	eLearning.mp.
21.	e–Learning.mp.
22.	mLearning.mp.
23.	m–Learning.mp.
24.	“virtual learning environment”.mp.
25.	1 or 18 or 19 or 20 or 21 or 22 or 23 or 24
26.	exp Education, Medical, Undergraduate/
27.	exp Education, Nursing/
28.	exp Medical Staff/
29.	exp Physicians/
30.	doctor?.mp.
31.	physician?.mp.
32.	exp Physician Assistants/
33.	exp Nurses/
34.	nurse?.mp/
35.	exp Nurses’ Aides/
36.	exp Allied Health Personnel/
37.	exp Community Health Workers/
38.	exp Health Personnel/
39.	exp Health Manpower/
40.	26 or 27 or 28 or 29 or 30 or 31 or 32 or 33 or 34 or 35 or 36 or 37 or 38 or 39
41.	25 and 40
42.	Randomized controlled trial.pt.
43.	Controlled clinical trial.pt.
44.	Randomized.ab.
45.	Placebo.ab.
46.	Drug therapy.fs.
47.	Randomly.ab.
48.	Trial.ab.
49.	Groups.ab.
50.	42 or 43 or 44 or 45 or 46 or 47 or 48 or 49
51.	exp animals/ not humans.sh.
52.	50 not 51
53.	41 and 52
54.	Limit 53 to yr = ”2000 –Current”Correspondence to:

*Source: Ovid MEDLINE® In_process& Other Non–Indexed Citations and Ovid MEDLINE® 1946 to Present. Date of search: 16 August 2013, 09:53. Limits: Year – 2000. Filter: Cochrane Highly Sensitive Search Strategy for identifying randomized trials in MEDLINE: sensitivity–maximizing version (2008 revision); Ovid format.

**Searching other resources.** We checked reference lists of the included studies and systematic reviews of the literature identified by our electronic searches for additional studies.

### Inclusion criteria

**Types of studies and participants.** We included studies published in any language on students of (i) undergraduate, health–related university degrees; or (ii) basic, health–related vocational training programmes. We defined undergraduate education or basic vocational training as any type of study leading to a qualification that: (i) is recognised by the relevant governmental or professional bodies of the country where the studies were conducted; and (ii) entitles the qualification–holder to apply for entry level positions in the health care workforce. For this reason, graduate medical education courses from the USA were included.

We considered studies on candidates for and holders of the qualifications listed in the Health Field of Education and Training of the International Standard Classification of Education (ISCED–F) [24], except studies on students of traditional and complementary medicine. We hence included students reading dental studies, medicine, nursing and midwifery, medical diagnostic and treatment technology, therapy and rehabilitation, or pharmacy. Medicine and dentistry were classified under the umbrella term *allied health professions*.

**Types of intervention.** First, we conducted a systematic mapping of the types of technologies used by the included studies to deliver the learning materials, through which we identified 6 broad categories of eLearning interventions, based on the technologies employed: (1) Offline computer–based eLearning, (2) Online and local area network–based eLearning, (3) Psychomotor skills trainer, (4) Virtual reality environments, (5) Digital game–based learning and (6) mLearning. We allocated each included study to the category that fitted the study best. Please refer to **Online Supplementary Document(Online Supplementary Document)** for a definition of these categories.

We only included studies in which online eLearning interventions were used to deliver the learning content, studies were categorized as online eLearning if the delivery of the learning content was done through the internet or intranet connections. Only studies that compared online eLearning or blended eLearning methods to: (i) traditional learning; (ii) an alternative eLearning or blended learning method; or (iii) no intervention was eligible for inclusion. These studies could either be studies where eLearning was the sole means by which the intervention was delivered or where eLearning was part of a complex, multi–component intervention.

**Types of outcome measures.** To be eligible for inclusion, studies had to report at least 1 of the following primary or secondary outcomes.

Primary outcomes: (1) Students’ knowledge, measured using any validated or non–validated instrument (eg, pre– and post–test scores, grades, perceived knowledge survey scores); (2) Students’ skills, measured using any validated or non–validated instrument (eg, pre– and post–test scores, time to perform a procedure, number of errors made whilst performing a procedure, perceived up–skilling); (3) Students’ satisfaction and attitudes towards eLearning, measured using any validated or non–validated instrument (eg, self–efficacy, satisfaction, acceptability).

Secondary outcomes: (1) Health economic properties of the interventions (eg, implementation cost, return on investment); (2) Adverse and/or unintended effects of eLearning (eg, potential feelings of depression and loneliness, dropout risks [25] and “computer anxiety” [26]).

We only considered studies to have measured students’ satisfaction and attitudes towards eLearning if they met all of the following criteria: (i) they compared the differences between intervention and control groups for these outcomes; (ii) the content of the survey questionnaires related to the teaching method (ie, eLearning method, blended learning, or traditional learning); and (iii) the adjectives used in the survey questionnaires accurately described attitudes and/or satisfaction.

### Study selection and data collection

**Figure 1:**
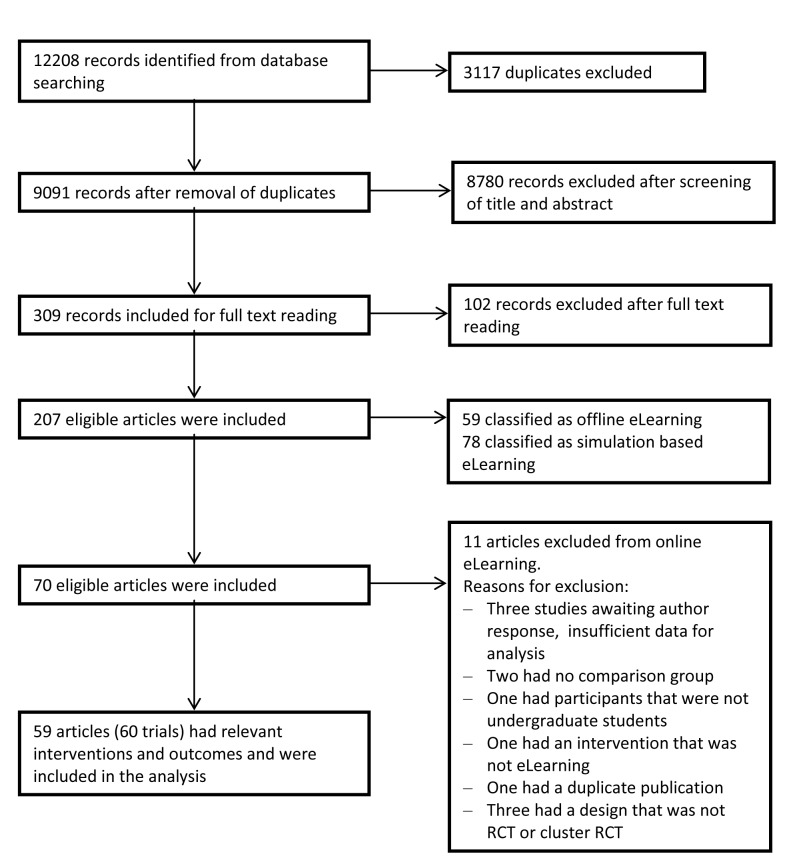
Flowchart of the studies included in the review.

The study selection process is summarised in the PRISMA flow diagram (**Figure 1**). In brief, we screened the titles and abstracts of the citations identified by our electronic and manual searches to identify potentially relevant studies, of which we assessed the full–text report to ensure they meet the inclusion criteria we specified. Review authors completed these tasks independently and met to compare their results and reach consensus.

Every selected study was allocated to a pair of review authors, with 10 review authors participating in total. Each review author independently extracted data from the included studies using the structured data extraction sheet shown in **Online Supplementary Document(Online Supplementary Document)**.

Each pair of reviewers compared their completed data extraction forms and any discrepancies between review authors’ results were resolved through discussion; if no agreement could be reached, a third review author acted as an arbiter. Because 10 review authors participated in the data extraction process, some categories were interpreted differently by some reviewers. Therefore, 3 reviewers went over the entire data extraction again to ensure uniformity.

We contacted authors of studies containing incomplete data to request the missing information. Some authors did not reply to our request for additional information, whilst other authors did not know the answer to our questions. For 1 study the response obtained from the author resulted in the subsequent exclusion of the study from the systematic review.

### Assessment of risk of bias in included studies

During the data extraction process, we assessed the risk of bias at the outcome level using tools recommended by the Cochrane Collaboration [23]. For RCTs, we did so across the domains of (1) random sequence generation, (2) allocation concealment, (3) blinding of participants and personnel, (4) blinding of outcome assessment, (5) incomplete outcome data, (6) selective outcome reporting, and (7) other bias including the comparability of intervention and control group; characteristics at baseline; validation of outcome assessment tools; reliability of outcome measures; and protection against contamination.

We assessed the risk of bias for cRCTs across the domains of (1) recruitment bias, (2) baseline imbalances, (3) loss of clusters and (4) incorrect analysis. For each study, 2 reviewers independently categorised each domain as low, high or unclear risk of bias.

### Summarising the data

We qualitatively compared the characteristics of the participants and of the interventions between the included studies to determine the feasibility of conducting a meta–analysis. Because of substantial clinical, educational, content and methodological heterogeneity we did not conduct a meta–analysis. Instead, we adopted a thematic summary approach [27].

## RESULTS

12 208 reports were identified from database screening of which 309 were retrieved for full–text evaluation of which 207 studies met the inclusion criteria (**Figure 1**). Fifty–nine articles [25–82] complied with the term internet and local area network or online eLearning (see **Online Supplementary Document(Online Supplementary Document)** for detailed description) and were included in the analysis. One study [83] involved students in 2 consecutive RCTs that were analysed separately (Ainsworth 2012A, Ainsworth 2012B). Thus the total number of evaluated trials was 60.

### Included studies

All studies were published in peer reviewed journals between 2000 and 2013 except 1 dissertation [81]. All included studies were parallel RCTs or cRCTs. The included number of RCTs and cRCTs suggests an increase in the number of publications after 2007 as eighteen of the included studies (30%) have been published between 2000 up and 2007 (ie, 8 years). The remaining 42 studies (70%) have been published in the shorter time interval between 2008 and mid 2013 (ie, 5.5 years). Out of all 60 included studies, 33 studies investigated eLearning in the field of medicine [26,28,32,34–36,38–41,44,45,49,50,54,58–62,64–70,72,75,77,78,80,82]. Eleven of the articles [25,27,33,42,43,53,56,74,76,79,83] were exclusively from nursing, 3 [55,57,81] were within the field of physical therapy, whereas 3 other studies within pharmacy [30, 31, 71]. Nine studies [29,37,46–48,51,63,73] investigated eLearning for dentistry students. Additionally, 1 article [52] dealt with medicine, nursing, and physical therapy while the remaining study recruited university students, but did not define their discipline [81].

### Participant characteristics

The total number of participants included across all trials was 6750 participants. The study with the smallest control group had 10 participants [77] whereas the largest control group had 249 participants [55]. The study with the smallest intervention group had 10 participants [33], while the largest intervention group had 349 participants [55]. Most studies were conducted among undergraduate university students apart from 9 studies [27,29–31,33,42,53,54,79] that investigated the effect of network–based eLearning for vocational training. Out of the 20 (33%) studies [30,32,33,40,45,47–49,52,53,55,57,64,65,71,73,75,76,83]that specified the age of the students, the lowest mean age of participants in a control group was 20.0 years [55] and the oldest was 30.0 years [76]. The lowest mean age in an intervention group was 19.9 years [75] and the highest was 30.0 years [76].

### Intervention characteristics

**Figure 2:**
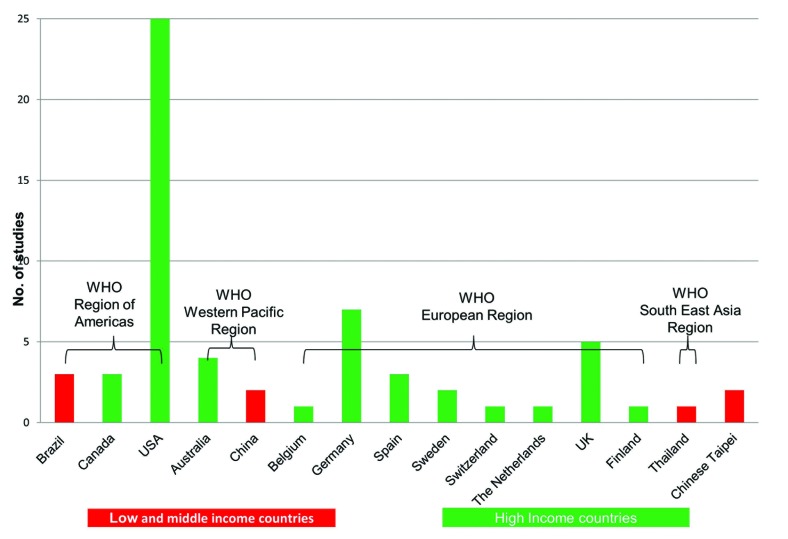
Country of origin of included, examined studies for low– and middle–income and high income countries separately.

Fifty studies compared eLearning to traditional learning, and 10 studies [26,35,36,45,46,61–63,66,74] compared 1 mode of eLearning to another mode of eLearning. The duration of exposure ranged from 9.05 minutes to 9 months [83]. Most of the studies (51 out of 60, 85%) were conducted exclusively in high income countries. Seven studies were conducted solely in low– to middle–income countries: 2 in Brazil [25,26]; 2 in China [38,56]; 1 in Thailand [33]; and 2 in the Chinese Taipei [27,42]. One study [52] was conducted simultaneously in Brazil and the USA (**Figure 2****).**

The majority of the studies used a website to present the learning material to the participants as part of their intervention [25–34,37–46,48–66,68–80,82,83]. Three studies [35,36,47] used a spaced education intervention, ie, an intervention in which the educational exposures are spaced and repeated over time [35]. In these studies the learning material was presented via email on a regular basis [35,36,47]. One study used video conference lectures as an intervention [67], and 1 study used a visual concept map [81].

### Primary outcomes

**Students’ knowledge assessment.** The knowledge gained from the exposure to the intervention was assessed in a number of different ways in the included studies. Overall, 53 [25–27,29–33,35–46,48–52,54,56–67,69,70,72–83] out of the 60 studies looked at a knowledge as an outcome. Nineteen of these studies [25,32,35,40,43,45,48–50,56,57,60,69,72,74,75,77,80,82] used only multiple choice questions (MCQs) to test students’ knowledge and understanding. Six studies used MCQs as a knowledge assessment tool together with an adaptive spaced test [36], gap text questions [51], matching and short answer questions [61], open ended and true/false questions [64], short essay questions [81] and a key features test [59].

Seven studies reported using test questions [31,39,40,44,67,70,78] to assess knowledge of study participants. Six other studies used open ended [30,33,63,66] or Likert type questionnaires [29] or even “fill in the blank” questions [73]. The rest of the studies measured students’ knowledge gain via other testing means including general numeracy tests [83]; written exams [41, 46, 58, 65]; independent observers’ assessment [79]; cognitive assessment instruments [54]; surgical knowledge test scores [76]; a Diagnostic Thinking Inventory and individual students’ performance in solving clinical reasoning problems [38]; a modified version of the Dartmouth Sleep Knowledge and Attitudes survey [62]; an interactive evaluation about melanoma [26]; an orthodontic examination form for each patient [37]; or some form of a knowledge assessment scale or checklist [42,52,68].

**Students’ skills assessment.** Skills were evaluated in 16 studies [28–30,32–34,39,42,53,55,57,63,68,69,71,72] using various methods to assess the outcome. Nine studies [33,34,42,55,63,68,71,72] used a rating scale and/or checklists (eg, an OSCE) to assess clinical skills. One study [53] used a search skills test, another 1 [29] a Likert type questionnaire while 3 studies evaluated students skills through written assessments such as data collection sheets [30], written case analysis [41] and open questions on standardised tasks [32]. Finally, 1 study [28] measured the degree of new skills acquisition by using a self–assessment report whilst another [39] measured the time that students made to complete the assigned exercise.

**Students’ satisfaction and attitudes towards eLearning.** Feedback from students assessed as their attitude towards the eLearning intervention was reported as an outcome in a total of 14 studies [28,29,32,33,43,45,48,49,54–56,63,64,72]. In all of these, students’ attitude was measured by questionnaires.

Student satisfaction was considered as an outcome in 33 studies [25,32–34,37–41,43,46–48,50–52,55,57–61,64–66,68,69,73–76,80,82]. Seventeen of these studies [33,34,38,40,51,52,55,57,59,61,64–66,68,73,75,76] mentioned that student satisfaction was evaluated with Likert scale questionnaires. The 16 remaining studies comparing student satisfaction among the students [25,32,37,39,41,43,46–48,50,58,60, 69,74,80,82] used different types of questionnaires or surveys without mentioning the use of Likert scales.

### Secondary outcomes

**Cost–effectiveness of the eLearning interventions.** Cost–effectiveness or cost–benefit or cost–utility of eLearning interventions were not assessed in any of the studies, however, some of the studies mentioned several financial and resource related elements of eLearning.

Buzzell et al. [54] mentioned that in the future many experts would be involved in content generation for their respective disciplines and that content could be shared online among their disciplines. Thus the online content development and delivery would not need the involvement of many faculty at all stages of content development and in turn educational institutions would be cost efficient. Stain et al. [67] mentioned that the costs of setting up videoconferencing were comarable to the costs of live lectures after an initial hardware investment of less than US$ 10 000. Stewart et al. [68] cited a paper saying that reduction of instructor training time, labour costs and institutional infrastructure could result in significant cost–efficiency. Toumas et al. [71] mentioned in the discussion that using the Internet leads to “reduced costs in terms of tutor–led workshops and is more efficient, enabling more complex topics to be covered in workshops”. Hauer et al. [34] deduced that the video cases were cheaper than the mini–CPX (Clinical Performance Evaluation) examination they used. An in–person examination of a class of 150 students cost approximately US$ 5400, which did not yet include Clinical Skills Centre maintenance costs, costs of case development and payment of Centre staff. In contrast, plain technologies as video cases were produced at a total cost of US$ 2200. Besides, the video cases could be reused freely, whereas the in–person mini–CPX requires annual purchase of a license.

In contrast, Fleming et al. [73] mentioned that the development of web–based or Computer Assisted Instruction is expensive in terms of time and energy spent. Phadtare et al. [52] made a general comment on the potential lack of necessary infrastructure and “new” costs associated with online courses.

**Adverse and/or unintended effects of eLearning.** Adverse or unintended events of the eLearning intervention were not reported in any of the studies.

### Excluded studies

Initially, 65 studies were categorised as online eLearning studies. We reclassified 2 studies [84,85] as non–networked computer–based because their eLearning interventions could be fully functional even without network technologies’ support. Three studies [86–88] were excluded because of insufficient data while another [35] was excluded as a duplicate paper. Seven studies [89–95] were excluded during the data extraction process, just before the analysis, because they met 1 or more of the exclusion criteria. Four of these 7 studies [91,92,94,95] were excluded because their study design was not a parallel or cRCT eg, a cross–over design [94]. Two studies [89,93] were excluded because they did not include comparison groups for the eLearning intervention eg, 2 different blended teaching methods using a common eLearning intervention in exactly the same way [93]. Finally, 1 study [90] used an eLearning intervention which was considered ineligible for our study ie, electronic voting during the lecture [90].

### Risk of bias in the included parallel RCTs

**Figure 3:**
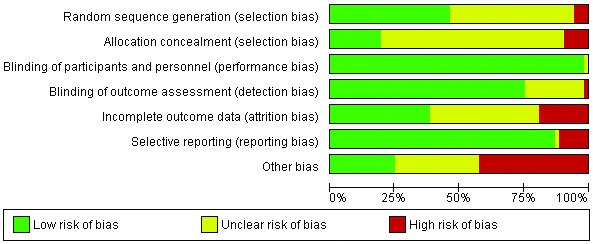
Overall risk of bias graph.

**Figure 4:**
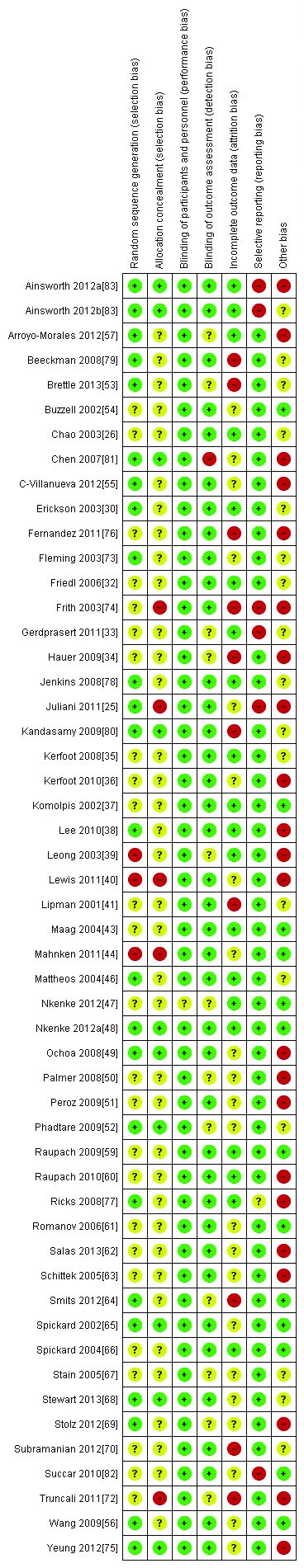
Risk of bias for each individual parallel randomised controlled trial (RCT) separately.

Thirty–one of the studies were considered to be at high risk of bias.[25,33,34,36,38–41,44,49–51,53,55,57,60,62–64,69,70,72,74–77,79–81,83]. Twenty–nine of the studies [26,30,32,35,43,48,52,54,56,59,61,65–67,73,78] had 1 or more categories classified as an unclear risk of bias, especially regarding the allocation of participants to intervention groups. There was only 1 study [47] with all the categories classified as low risk of bias (**Figure 3**** and ****4**). Risk of bias is described in detail in the **Online Supplementary Document(Online Supplementary Document)**.

### Effects of online eLearning interventions

The 60 randomized trials included in our review assessed the effectiveness of online eLearning interventions in terms of knowledge, skills, attitudes and satisfaction. The findings were based on comparisons between online eLearning and traditional learning or between various modes of online eLearning. A study may have compared more than 1 outcome between groups, and each outcome may have been assessed in multiple ways. For example, a study which compared students’ acquisition of skills may have assessed skills in terms of the student’s performance on a global rating scale, the ability to perform a specific procedure, as well as the ability to comply with the requirements in a checklist. As a result, the number of comparisons made across studies for a particular outcome may exceed the number of studies which reported on that outcome. Only 2 studies [32,33] measured all specified outcomes of knowledge, skills, attitudes and satisfaction.

The studies were split into 2 research themes evaluating the impact of eLearning interventions for undergraduate health care education: traditional learning vs online eLearning, and online eLearning vs online eLearning.

### Traditional learning vs online eLearning

Fifty of the 60 included studies (83%) compared online eLearning with traditional learning. [25,27–34,37–44,47–58,60,64,65,67–73,75–83]. **Table 2** presents the summary of the findings of the individual studies. Further description of the nature of the interventions is in the **Online Supplementary Document(Online Supplementary Document)**.

**Table 2:** Summary of findings from the 50 studies which compared online eLearning with traditional learning

Study	Discipline	Knowledge	Skills	Attitude	Satisfaction	No. of participants	Intervention delivery approach	Characteristics
Ainsworth 2011a [83]	Nursing	NS						
Ainsworth 2011b [83]	Nursing	E						
Arroyo–Morales 2012 [57]	Physiotherapy	NS	E		NS	46	Blended learning	IG: Online tutorial (ECOFISIO) CG: Self–study
Baumlin 2000 [58]	Medicine	NS			NS	100	Blended learning	IG: Computer Assisted Instructions (EMCyber–School) CG: Lectures
Beeckman 2007[79]	Nursing	E				426	Full eLearning	IG: 1 h eLearning program CG: 1 h lecture
Brettle 2013 [53]	Nursing		NS			77	Full eLearning	IG: Online tutorial CG: 1 h face–to–face tutorial
Buzzell 2002 [54]	Medicine	NS		NS		34	IG1: Full eLearning IG2: Blended learning	IG: Web–based tutorials IG2: Traditional lectures with web–based tutorials CG: Traditional lectures
Cantarero2012 [55]	Physiotherapy		NS	DNT	DNT	50	Full eLearning	IG: Online materials CG: access to books and documents
Chen 2007 [81]	Undefined	NS				145	IG1: Full eLearning IG2: Traditional learning	IG1: A visual advance organizer IG2: Text outline CG: Textbook reading without an advance organizer (AO)
Chen 2012 [27]	Nursing	M				146	Blended learning	IG: Online tests CG: Group A: Paper references, Group B: No assistance
Cox 2008 [28]	Medicine		DNT	DNT		138	IG1: Traditional learning IG2: Blended learning	IG1: Group discussion IG2: Website, Videos CG: Paper based, traditional learning materials
DeBate 2013 [29]	Dentistry	T	DNT	NS		608	Full eLearning	IG: Online (computer and website) CG: Regular curriculum
Erickson 2003 [30]	Pharmacy	M	M			42	IG1: Traditional learning IG2: Full eLearning	IG1: Lecture–based tutorial IG2: Web–based tutorial CG: No intervention
Fernandez 2011[76]	Nursing	NS			NS	116	Full eLearning	IG: Computer assisted learning CG: Face–to–Face lecture
Fleming 2003 [73]	Dentistry	NS			NS	31	eLearning and traditional learning separately	IG: Slide/audiotape self–instruction and web–based self–instruction CG: Web–based self–instruction and slide/audiotape self–instruction
Flowers 2010 [31]	Pharmacy	E				79	Blended learning	IG: Web–based Multimedia Vignettes CG: No Web–based Multimedia Vignettes
Friedl 2006 [32]	Medicine	NS	DNT	NS	NS	126	Full eLearning	IG: online multimedia course CG: Text books
Gerdprasert 2010 [33]	Nursing	NS	E	NS	NS	84	Blended eLearning	IG: Web, interactive graphics, animation CG: Traditional teaching
Hauer 2013 [34]	Medicine		E		E	303	IG1: Full eLearning IG2: Traditional learning	IG: Web based module CG: Group work, role play.
Jenkins 2008 [78]	Medicine	NS				73	Blended learning	IG: Internet–based tutorial CG: Lecture.
Juliani 2011[25]	Nursing	NS			DNT	80	Full eLearning	IG: Designing a schedule with internet CG: Designing a schedule without internet
Kandasamy 2009 [80]	Medicine	T			NS	62	Full eLearning	IG: Online CAI module CG: Review articles (Text based)
Komolpis 2002 [37]	Dentistry	NS			NS	99	Full eLearning	IG: Digital records on PC CG: Hardcopy records
Lee 2010 [38]	Medicine	NS			NS	52	Blended learning	IG: Web–based problems, workshop CG: No workshop
Leong 2003 [39]	Medicine	M	NS		NS	54 vs 325*	IGI: Full eLearning IG2: Full eLearning	IG1: Computer based cases (other than C1+C2) IG2: Computer based cases (C1+C2) CG: No Computer based cases
Lewis 2011 [40]	Medicine	T			NS	39	Blended learning	IG: Web–based MCQ's CG: Textbook resource
Lipman 2001[41]	Medicine	E			NS	130	Blended learning	IG: Website, books CG: Books, discussions
Lu 2009 [42]	Nursing	NS	E			147	Blended learning	IG: Lectures and interactive web–based course CG: lectures only
Maag 2004[43]	Nursing	NS		NS	NS	96	IG1: Traditional learning IG2: Blended learning IG3: Blended learning	IG1: Text and image IG2: Text and image and animation IG3: Text, Image, Animation, and Interactivity CG: Text modules
Mahnekn 2010[44]	Medicine	NS				96	IG1: Blended learning IG2: Blended learning	IG1: eLearning, self IG2: eLearning, mandatory CG: No access to eLearning
Nkenke 2012 [48]	Dentistry	NS		NS	NS	42	Blended learning	IG: Technology enhanced learning CG: Didactic lectures, PowerPoint presentation
Nkenke 2012[47]	Dentistry				NS	42	Blended learning	IG: Spaced education CG: Lectures
Ochoa 2008 [49]	Medicine	E		NS			Full eLearning	IG: Web–based interactive program CG: Traditional text.
Palmer 2008 [50]	Medicine	NS			DNT	130	IG1: Traditional learning IG2: Blended learning IG3: Blended learning	IG1: Written case–studies IG2: Clinical material + interactive computer–based case studies IG3: Clinical material + interactive computer–based case studies
Peroz 2009 [51]	Dentistry	E			NS	85	Blended learning	IG: Online education CG: PowerPoint, discussions
Phadtare2009 [52]	Medicine; Nursing; Physiotherapy	E			E	48	Full eLearning	IG: Online materials CG: Off–line materials
Raupach 2009 [59]	Medicine	NS			DNT	148	Blended learning	IG: Web–based teaching module CG: face–to–face traditional lecture
Raupach 2010 [60]	Medicine	E			E	74	Blended learning	IG: Web–based module CG: Traditional lecture
Ricks 2008 [77]	Medicine	E				23	Full eLearning	IG: Computer Assisted Learning group CG: No intervention
Smits 2012 [64]	Medicine	NS		T	NS	128	Full eLearning	IG: Case based eLearning CG: Text based learning
Spikard 2002 [65]	Medicine	NS			E	95	Full eLearning	IG: Online lecture CG: Traditional learning
Stain 2005 [67]	Medicine	NS				12 vs 98*	Full eLearning	IG: Videoconference lectures CG: Conventional lectures
Stewart 2013 [68]	Medicine		DNT		NS	71	Blended learning	IG: Online access to learning content CG: Standard content
Stolz 2012 [69]	Medicine	NS	T		T	129	Full eLearning	IG: Web–based training CG: Lectures
Subramanian 2012 [70]	Medicine	E				33	Full eLearning	IG: Interactive medical software CG: Lectures
Succar 2010 [82]	Medicine	DNT			NS	147	Blended learning	IG: Computer based training CG: Traditional teaching
Toumas 2009 [71]	Pharmacy		E			236	Blended learning	IG: Internet–based Tutorial CG: Small group workshop
Truncali 2011 [72]	Medicine	E	E	DNT		141	Blended learning	IG: Web–based Tutorial CG: Lectures
Wang 2009 [56]	Nursing	E		NS		133	Blended learning	IG: Online, self–learning CG: Traditional multimedia lecture
Yeung 2013 [75]	Medicine	NS			NS	78	Blended learning	IG: Computer–assisted learning CG: Text–/image–based learning (TBL)

E – Results favoured online eLearning over traditional learning, NS – No significant difference between online eLearning and traditional learning, MCQ – Multiple choice questions M – Mixed results, T – Results favoured traditional learning over online eLearning, DNT – Difference not tested, CG – Control group, IG – Intervention group

*Average number students exposed to videoconference lectures and conventional lectures, respectively.

**Students’ knowledge.** Amongst the 60 studies which compared online eLearning with traditional learning, knowledge was assessed in 43 RCT studies (86%) [25,27,29–31,37–44,48–52,54,56–60,64,65,67,69,70,72,73,75–83] and 7 cRCT studies [27–29,31,42,58,71].

Twelve studies (27%) assessing knowledge gain demonstrated significantly higher knowledge gains for students assigned to the online eLearning compared to those exposed to traditional learning [31,41,49,51,52,56,60,70,72,77,79,83]. Outcome measures for these studies were based on test items or questions [31, 70], written case analyses [41], MCQs [50,51,56,60,72,77], the Six–subgroup Quality Scale (SSQS) [52], a general numeracy test [83] and independent assessments by evaluators [79]. The sample size of these studies ranged from 39 to 1475. Six of these 12 studies were conducted on medical students [41,49,60,70,72,77], 3 among nursing students [56, 79, 83], 1 among dentistry students [51], 1 among pharmacy students [31], while 1 study [52] was conducted among medicine, nursing, and physical therapy students. Five of these studies used full online eLearning as the main intervention [49, 52, 70, 77, 79] whereas 7 used blended learning [31,41,51,56,60,72,83].

Post–intervention knowledge was not significantly different between eLearning and traditional learning in 24 (48%) of the included studies [25,32,33,37,38,42–44,48,50,54,57–59,64,65,69,73,75,76,78,81,83]. Three studies [27, 30, 39] showed mixed results ie, favouring the intervention, control or neither 1 depending on the specific indicator of knowledge being assessed. In 1 (2%) study [82] knowledge was assessed but not tested for statistically significant differences between the intervention groups.

Finally, there were 3 studies [29,30,80] that demonstrated significantly higher knowledge gains for students assigned to traditional learning compared to those exposed to online eLearning. Two of these studies [29,80] used full online eLearning as the main intervention while the other 1 [40] used blended learning.

**Students’ skills.** Overall, 15 studies – 11 RCTs [30,32–34,39,53,55,57,68,69,72] and 4 cRCTs [28,29,42,71] measured skills as outcome.

Of the studies that evaluated differences in skill acquisition, 6 (40%) found significantly greater skill acquisition amongst students assigned to online eLearning [33,34,42,57,71,72]. The number of participants included in these studies ranged from 44 to 303. Two of these studies were conducted in medical students [34,72], 2 in nursing students [33,42], 1 in physiotherapy students [57] and 1 in pharmacy students [71]. Four of these studies used traditional learning as their main intervention [34,42,57,71], whereas 2 used blended learning as the main intervention [33,72].

Three studies (21%) did not detect a significant difference in skill acquisition between groups [39,53,55]. One study [30] showed mixed results ie, favouring the online eLearning or the traditional learning group depending on the specific indicator of skills being assessed. This study had 3 groups, comparing pharmacy students’ knowledge and ability to assess metered–dose inhaler (MDI) after a lecture based tutorial, a web–based tutorial and being provided no teaching on the topic at all. Finally, there was 1 study [69] that demonstrated significantly higher skill gains for students assigned to traditional learning compared to those exposed to online eLearning. This study used full eLearning as the main intervention.

**Students’ attitudes and satisfaction.** Twelve studies (24%) – 10 RCTs [32,33,43,48,49,54–56,64,69,72] and 2 cRCTs [28, 29] – assessed attitudes as an outcome of the intervention through questionnaires.

Eight of these studies [29,32,33,43,49,50,54,56] (67%) did not find a statistically significant difference between the 2 types of learning methods, or the study showed mixed results for online eLearning vs traditional learning depending on the test evaluated. Three studies [28,55,72] assessed attitude, but did not test for statistically significant differences between the intervention groups. None of the studies reported a significant result on student attitudes favouring online eLearning interventions.

The remaining study (8%) [64] reported more positive attitudes towards the intervention in the traditional learning groups. This study used full online eLearning as the main intervention.

Student satisfaction was assessed in 28 RCTs [25,32–34,37–41,43,47,48,50–52,55,57,59,60,64,65,68,69,73,75,76,80,82] and 1 cRCT [58].

Out of 29 studies looking at the level of student satisfaction, 4 [34,52,60,65] (14%) found a significantly greater proportion of students exposed to online eLearning who were satisfied compared to those exposed to traditional learning. One of these 4 studies [60] compared blended learning with traditional learning, whilst the other 3 [34,52,65] used full eLearning interventions compared with traditional learning ones. Twenty studies (74%) did not detect any significant difference [32,33,37–41,43,47,48,51,57,58,64,68,74–76,80,82] while in 4 studies satisfaction was assessed [25,50,55,59] but not tested for statistically significant differences between the intervention groups.

There was 1 study [69] using full online eLearning as the main intervention that reported a statistically significant better student satisfaction in the traditional learning group.

### Comparison of different types of eLearning against each other

Ten (18%) of the included studies [26,35,36,45,46,61–63,66,74] compared the effectiveness of various modes of online eLearning against each other. Eight of these studies [26,35,45,46,61–63,74] compared groups of eLearning with different levels of student interaction. In 2 of them “interactivity” was also facilitated by collaborative tools, ie, online web chats [74] and discussion forums and online message systems [61].

**Students’ knowledge.** All of the 10 studies [26,35,36,45,46,61–63,66,74] comparing various forms of online eLearning measured and reported their effects on knowledge.

Five studies observed a difference in results between different modalities of eLearning. In a study comparing an adaptive form of spaced education against a linear, repetitive one [36], the adaptive eLearning intervention showed better results than its “passive” form. Another study showing significant knowledge acquisition for an “active” eLearning intervention was Chao et al [26] where a linear educational environment (website) supported by complementary information (skin anatomy images) which users could access at will was compared to a non modified website. Similarly, in 1 study [63] an eLearning intervention, allowing students to play a video back and forth at their will showed better knowledge gains in comparison to an eLearning intervention where the procedure was linear. In a study [61] on a “passive” type of eLearning, offering course material through conventional World Wide Web technology and by letting students engage with the instructor only by email resulted in higher knowledge gains in comparison to an interactive eLearning intervention where students could make use of all the learning tools of the Web CT (online proprietary virtual learning environment system) [61]. A “passive” eLearning intervention showed favourable results also for Salas et al [62]. In this study, participants in the “passive” eLearning group were solely provided with a list of random sleep facts and trivia presented in a PowerPoint format. The “active” eLearning intervention consisted of an online, self–paced, sleep medicine learning module.

Non statistical significant differences were found in 4 studies [35,45,66,74] comparing different online eLearning modalities. One study showed no difference in knowledge acquisition between eLearning modes [46].

**Students’ skills.** Skill acquisition was assessed in 1 study [63]. This study showed no significant differences in skills acquisition between the 2 different (active vs passive) eLearning modalities.

**Students’ attitudes and satisfaction.** Manikam et al. [45] and Schittek Janda et al. [63] were the only studies amongst the 10 studies comparing different eLearning modalities that assessed attitude. The study by Manikam et al showed no difference in students’ attitudes between the 2 eLearning modes. In this study a dummy learning package was compared to the ABD learning package, ie, symptom–based decision–making pathways software. Schittek Janda et al. reported no significant differences in skills acquisition between the 2 different (active vs passive) eLearning modalities.

Four (40%) studies [46,61,66,74] compared the effects of different eLearning modes on student satisfaction. Two studies [46,61] showed no difference in students’ satisfaction for the 2 eLearning modes. Frith et al. [74] reported that students in the group that used collaboratively a 6–week Web–based course on cardiac rhythm interpretation supported by online chat software were more satisfied than students in the group who worked on the same course independently. In the study by Spickard et al. [66] students in the groups of the online lecture of power point slide presentation with audio narration were more satisfied than students in the group of the online lecture of power point slide presentation without audio narration.

## DISCUSSION

### Summary of main results

This systematic review reports on the effectiveness of online eLearning for undergraduates in health professions. We found that online eLearning does lead to changes in knowledge, skills, attitude and satisfaction and seems to be more effective than traditional learning in terms of knowledge and skills gained. Our findings are similar to previous reviews of online eLearning [21,22,96–100] and offline eLearning [18].

In our review, 29% of the studies showed significantly higher knowledge gains, 40% of the studies showed significantly greater skill acquisition, 67% of the studies showed no difference in attitude and 14% of the studies showed higher satisfaction with online eLearning than traditional learning. The participants in the included studies were from the fields of medicine, dentistry, pharmacy or medical allied studies enrolled at universities, with a smaller number conducted at vocational training centres or colleges. Consequently, the results of this systematic review apply to students from similar disciplines, universities and colleges. The majority of the studies were conducted in high–income countries with exception of few [25,26,33,38,42,52,56,101] which were from low to middle income countries, hence these results are generalizable only to their corresponding settings.

The studies included in our review had a high degree of methodological, educational and clinical heterogeneity, similar to previous reports [21,22,96–100]. Knowledge assessment was, for example, conducted using different test items or questions [31,70], written case analyses [41], MCQs [49,51,56,60,72,77] the Six–subgroup Quality Scale (SSQS) [52], a general numeracy test [83] and independent assessments. Similarly, there was variability in the assessment of skills, attitudes and satisfaction across the studies. Hence pooling of effect estimates was not possible. Mode of online interventions varied across the studies, most of the studies used a website, while some used other interventions such as spaced education, video lectures or visual concept map. Furthermore, there were great variations in exposure time to the eLearning intervention. Financial and resource related elements of eLearning was reported only in 8 studies [34,52,54,59,67,68,71,73]. Nevertheless, none of the studies included a robust cost–effectiveness analysis of eLearning vs traditional learning and therefore it is not possible to provide an assessment on cost–effectiveness of online eLearning. Furthermore, no studies reported on the adverse effects of online eLearning.

The overall quality of evidence included in this systematic review is not uniform and contains a significant number of studies with methodological weaknesses with only 1 high quality study [48]; similar findings were reported in previous reviews. [16,19,20,22,102,103] Most of the included studies did not adhere to the CONSORT guidelines for reporting of RCTs [104] and thus their risk of bias was unclear. Several of the included studies had high risk of bias due to volunteer [49,51,57,60,72,74,83] and attrition bias. [34,41,70,72,79]. Due to the weaknesses of most of the included studies a strong conclusion on whether there is a clear difference between online eLearning and traditional learning effectiveness that applies to the general population of learners cannot be drawn.

Our study has many strengths. The review was based on a thorough search of available literature which identified a large number of potentially eligible studies identified and synthesized by a multi–disciplinary international team and it offers a number of advantages over previous work in this area. The key strength is an attempt to combine breadth of scope in terms of widely defining eLearning and the range of health professions covered. The review encompasses all empirical studies (RCTs). To ensure data quality, article screening and data extraction was done independently by 2 persons to avoid subjective bias, disagreements were resolved through discussion. The review included studies from both developed and developing countries and thus provides crucial information on the usage, effectiveness and applicability of online eLearning in these settings. Finally, the review used standard methods for systematic reviews and meta–analyses in accordance with preferred reporting items for systematic reviews and meta–analyses (PRISMA) which makes it transparent.

The review had a few limitations. The included studies had several methodological rudiments, we contacted the authors to obtain necessary information for assessing the risk of bias for these studies, however due to time constraints, and it was not possible to contact all authors. Moreover, due to the lack of a uniform, standardized terminology for eLearning studies, we categorized studies as online eLearning (ie, local area network or web–based) and offline eLearning (ie, non–networked or computer based). Although we assigned each individual study to only 1 category, it is important to highlight that there might be some degree of overlap between categories as 1 form of technology may be built on another one.

In summary, this systematic review compares online eLearning and traditional learning in undergraduate health–related students and consolidates current knowledge on the effectiveness of online eLearning. The evidence from the highest and the lowest quality studies indicates that online eLearning is equivalent to and perhaps even more effective than traditional learning in terms of knowledge and skills gained. The generalization of these findings is limited only to the studied population in the review.

Online learning’s ubiquity provides a convenient and possibly a more cost–effective alternative to traditional learning and has great potential in supporting health care workforce capacity building and competency development globally. This review highlights the need for improvements in the methodological design in future studies.

### Implications for policy makers

The findings of this review present a potential incentive for policy makers to encourage adoption and the development of online eLearning programs. These online eLearning programs could be useful in training health care professionals in countries with acute health care worker shortage, without substantial investments. These online technologies if adopted earlier could help lower the burden of diseases by increasing the health care professional per capita. Though adoption of these online technologies would involve some initial start–up cost, it would be largely beneficial as the potential for the return on investment is high in terms of health gains and lives saved.

### Implications for educational institutions

Online eLearning offers many opportunities. This review shows that eLearning is as effective as the traditional learning and with many advantages compared to traditional learning. The universities could adopt these technologies and could reach out to a wider audience within and outside their country, thus offering a tremendous growth opportunity for the educational institutions. Institutions could employ online eLearning to train their health workforce without having to spend for their travel elsewhere within or outside their countries.

### Implications for future research

The findings of the review have many implications for research. Future evaluations of online eLearning should aim to answer many remaining research questions from intervention design features to setting or modality for online eLearning, and build cost–effectiveness models. We should especially aim to strengthen the evidence base for developing countries.
