# Supplementary material for: Online eLearning for undergraduates in health professions: A systematic review of the impact on knowledge, skills, attitudes and satisfaction
Source: J Glob Health. 2014 Jun;4(1):010406. doi: 10.7189/jogh.04.010406 (PMC4073252; doi:10.7189/jogh.04.010406)
Supplement: Online Supplementary Document [file jogh-04-010406-s001.pdf]

Online Supplementary Document

George et al. Online eLearning for undergraduates in health professions: A systematic review of the impact on knowledge, skills, attitudes and satisfaction
J Glob Health 2014;4:010406

1: Definition of the six eLearning categories

Six broad categories of eLearning interventions, based on the technologies employed. The categories were defined as follows:

• Offline computer-based eLearning: standalone applications where internet or intranet connections are not required for the delivery of the learning activities. The main tasks of the eLearning software in this category are usually performed on a PC or laptop. The delivery channels are usually CD-ROM or USB memory sticks. Alternatively, the delivery mode of the software can be via a networked connection, as long as the learning activities do not rely on this connection.

• Online and local area network-based eLearning: interventions that use the transmission control protocol (TCP) and the internet protocol (IP) to provide the full functionalities of the educational intervention. As implied by the terminology used, the delivery channels are usually the internet or a local area network.

• Psychomotor skills trainer: technology that will develop fine motor coordination skills and techniques in education, such as the precise use of instruments of tools.
• Virtual reality environments: computer-generated representations of a real or artificial environment. This can be interacted with by external involvement, allowing for a first-person active learning experience.

• Digital game-based learning: the application of game principles and mechanics in non-game contexts to engage users in solving problems and improve their engagement, attitudes, motivation and knowledge.

• mLearning: any eLearning intervention that uses handheld, mobile devices to deliver educational content such as a mobile phone, iPod or tablet.

2: Fields included in the data extraction form

1. Study ID

2.1. Journal where the study was published

2.2. Type of publication

2.3. Authors' affiliation

3.1. Study design as specified in the report

3.2. Study aims & objectives

3.3. Countries where the study was conducted

3.4. WHO region

3.5.World Bank income category

3.6. Study start date

3.7. Study end date

3.8. Method of comparison

4.1. Total number of participants invited to take part in the study

4.2. Total number of participants who agreed to take part in the study

4.3. Total number of participants meeting the inclusion criteria for participation in the study

4.4. Total number of participants included in the study

4.5. If cluster RCT, total number of clusters initially included in the study

4.6. If cluster RCT, total number of clusters randomised

4.7. Inclusion criteria

4.8. Exclusion criteria

5.1. Total number of experimental groups (including the control group)

5.2. Were groups tested for baseline differences?

5.2.1. If there were baseline differences, please specify what the difference was

5.3. Indicate the type of degree or qualification that participants were pursuing

If other, please specify:

5.4. Year of study within the anticipated degree or qualification

5.5. Control group

5.5.1. Total number of participants/clusters allocated to the control group

5.5.2. Mean age (standard deviation) of the participants in the control group

5.5.3. Name of educational intervention used as control

5.5.4. Description of the control condition

5.5.5. Field of study

5.6.6. Exposure to the control condition during the whole study

5.5.7. Total exposure time to the intervention

5.5.8. Type of technology/devices used to deliver the intervention

5.5.9. Delivery approach of the intervention

If other, please specify:

5.5.10. Was the usual delivery mode of the assessment changed?

5.5.11. If yes, please specify

5.5.12. Was the delivery mode of the assessment uniform across all the experimental groups?

5.6. Intervention group I

5.6.1. Total number of participants/clusters allocated to this intervention group.

5.6.2. Mean age (standard deviation) of the participants in this intervention group

5.6.3. Name of educational intervention used in this intervention group

5.6.4. Description of this intervention condition

5.6.5. Field of study

5.6.6. Exposure to this intervention condition during the whole study

5.6.7. Total exposure time to the intervention

5.6.8. Type of technology/devices used to deliver the intervention

5.6.9. Delivery approach of the intervention

If other, please specify:

5.6.10. Was the usual delivery mode of the assessment changed?

5.6.11. If yes, please specify

5.6.12. Was the delivery mode of the assessment uniform across all the experimental groups?

5.7. Intervention group II

5.7.1. Total number of participants/clusters allocated to this intervention group.

5.7.2. Mean age (standard deviation) of the participants in this intervention group

5.7.3. Name of educational intervention used in this intervention group

5.7.4. Description of this intervention condition

5.7.5. Field of study

5.7.6. Exposure to this intervention condition during the whole study

5.7.7. Total exposure time to the intervention

5.7.8. Type of technology/devices used to deliver the intervention

5.7.9. Delivery approach of the intervention

If other, please specify:

5.7.10. Was the usual delivery mode of the assessment changed?

5.7.11. If yes, please specify

5.7.12. Was the delivery mode of the assessment uniform across all the experimental groups?

5.8. Intervention group III

5.8.1. Total number of participants/clusters allocated to this intervention group.

5.8.2. Mean age (standard deviation) of the participants in this intervention group

5.8.3. Name of educational intervention used in this intervention group

5.8.4. Description of this intervention condition

5.8.5. Field of study

5.8.6. Exposure to this intervention condition during the whole study

5.8.7. Total exposure time to the intervention

5.8.8. Type of technology/devices used to deliver the intervention

5.8.9. Delivery approach of the intervention

If other, please specify:

5.8.10. Was the usual delivery mode of the assessment changed?

5.8.11. If yes, please specify

5.8.12. Was the delivery mode of the assessment uniform across all the experimental groups?

If more than 4 intervention groups (including the control group), please copy and paste the relevant cells as needed

6.1. Was 'Knowledge' measured? - If not, please go to section 6.2.

6.1.1. Instrument or measure used to asses knowledge - as specified by the study authors

6.1.2. Is this a validated instrument?

6.2. Were 'Skills' measured? - If not, please go to section 6.3.

6.2.1. Instrument or measure used to asses skills - as specified by the study authors

6.2.2. Is this a validated instrument?

6.3. Were 'Attitudes' measured? - If not, please go to section 6.4.

6.3.1. Instrument or measure used to asses attitudes - as specified by the study authors

6.3.2. Is this a validated instrument?

6.4. Was 'Student satisfaction' measured? - If not, please go to section 6.5.

6.4.1. Instrument or measure used to asses student satisfaction - as specified by the study authors

6.4.2. Is this a validated instrument?

6.5. Was an economic evaluation of the eLearning intervention performed?

6.5.1. Were quantitative indicators like costs, investments, hardware, software, license fees and benefits/savings of the eLearning intervention measured?

6.5.2. Was the urgency of the eLearning intervention (i.e., due to a new regulation or organisational demand) mentioned?

6.5.3. Were qualitative-strategic indicators of the eLearning intervention like quality and performance improvements measured?

6.5.4. Were external factors of the eLearning intervention like synergy effects or economies of scope measured?

6.5.5. Please list any additional economic indicators that were measured

7.1. Selection bias

7.1.1. Random sequence generation

7.1.1.1. Describe the method used to generate the allocation sequence in sufficient detail to allow an assessment of whether it should produce comparable groups

7.1.1.2. Please indicate your judgement

7.1.2. Allocation concealment

7.1.2.1. Describe the method used to conceal the allocation sequence in sufficient detail to determine whether intervention allocations could have been foreseen in advance of, or during, enrolment

7.1.2.2. Please indicate your judgement

7.2. Performance bias

7.2.1. Blinding of participants and personnel

7.2.1.1. Describe all measures used, if any, to blind study participants and personnel from knowledge of which intervention a participant received. Provide any information relating to whether the intended blinding was effective

7.2.1.2. Please indicate your judgement

7.3. Detection bias

7.3.1. Blinding of outcome assessment

7.3.1.1. Describe all measures used, if any, to blind outcome assessors from knowledge of which intervention a participant received. Provide any information relating to whether the intended blinding was effective

7.3.1.2. Please indicate your judgement

7.4. Attrition bias

7.4.1. Incomplete outcome data

7.4.1.1. Describe the completeness of outcome data for each main outcome, including attrition and exclusions from the analysis. State whether attrition and exclusions were reported, the numbers in each intervention group (compared with total randomized participants), reasons for attrition/exclusions where reported, and any re-inclusions in analyses performed by the review authors

7.4.1.2. Please indicate your judgement

7.5. Reporting bias

7.5.1. Selective reporting

7.5.1.1. State how the possibility of selective outcome reporting was examined by the review authors, and what was found.

7.5.1.2. Please indicate your judgement

7.6. Other bias

7.6.1. Other source of bias

7.6.1.1. State any important concerns about bias not addressed in the other domains in the tool

7.6.1.2. Please indicate your judgement

8.1. Recruitment bias

8.1.1. Please describe any evidence of recruitment bias.

8.2. Baseline imbalances

8.2.1. Please describe any evidence of baseline imbalances.

8.3. Loss of clusters

8.3.1. Please indicate any evidence of risk of bias due to loss of clusters.

8.4. Incorrect analysis

8.4.1. Please indicate any evidence of incorrect analysis.

9.1. Control group

9.1.1. Outcome reported

9.1.2. Measure of effect size (as measured by the study authors)

9.1.3. Measure of dispersion (as measured by the study authors)

9.1.1. Outcome reported

9.1.2. Measure of effect size (as measured by the study authors)

9.1.3. Measure of dispersion (as measured by the study authors)

9.1.1. Outcome reported

9.1.2. Measure of effect size (as measured by the study authors)

9.1.3. Measure of dispersion (as measured by the study authors)

9.1.1. Outcome reported

9.1.2. Measure of effect size (as measured by the study authors)

9.1.3. Measure of dispersion (as measured by the study authors)

If more than one outcome was reported, please insert more cells here and copy and paste the relevant data entry boxes.

9.2. Intervention I group

9.2.1. Outcome reported

9.2.2. Measure of effect size (as measured by the study authors)

9.2.3. Measure of dispersion (as measured by the study authors)

9.2.1. Outcome reported

9.2.2. Measure of effect size (as measured by the study authors)

9.2.3. Measure of dispersion (as measured by the study authors)

9.2.1. Outcome reported

9.2.2. Measure of effect size (as measured by the study authors)

9.2.3. Measure of dispersion (as measured by the study authors)

9.2.1. Outcome reported

9.2.2. Measure of effect size (as measured by the study authors)

9.2.3. Measure of dispersion (as measured by the study authors)

If more than one outcome was reported, please insert more cells here and copy and paste the relevant data entry boxes.

9.3. Intervention II group

9.3.1. Outcome reported

9.3.2. Measure of effect size (as measured by the study authors)

9.3.3. Measure of dispersion (as measured by the study authors)

9.3.1. Outcome reported

9.3.2. Measure of effect size (as measured by the study authors)

9.3.3. Measure of dispersion (as measured by the study authors)

9.3.1. Outcome reported

9.3.2. Measure of effect size (as measured by the study authors)

9.3.3. Measure of dispersion (as measured by the study authors)

9.3.1. Outcome reported

9.3.2. Measure of effect size (as measured by the study authors)

9.3.3. Measure of dispersion (as measured by the study authors)

If more than one outcome was reported, please insert more cells here and copy and paste the relevant data entry boxes.

9.4. Intervention III group

9.4.1. Outcome reported

9.4.2. Measure of effect size (as measured by the study authors)

9.4.3. Measure of dispersion (as measured by the study authors)

9.4.1. Outcome reported

9.4.2. Measure of effect size (as measured by the study authors)

9.4.3. Measure of dispersion (as measured by the study authors)

9.4.1. Outcome reported

9.4.2. Measure of effect size (as measured by the study authors)

9.4.3. Measure of dispersion (as measured by the study authors)

If more than one outcome was reported, please insert more cells here and copy and paste the relevant data entry boxes.

9.5. Comparison I

9.5.1. Please indicate the intervention groups being compared

9.5.2. Please indicate the outcomes being compared

9.5.3. Statistical test used for the comparison

9.5.4. Result of the test

9.5.5. P value / Confidence intervals

9.6. Comparison II

9.6.1. Please indicate the intervention groups being compared

9.6.2. Please indicate the outcomes being compared

9.6.3. Statistical test used for the comparison

9.6.4. Result of the test

9.6.5. P value / Confidence intervals

9.7. Comparison III

9.7.1. Please indicate the intervention groups being compared

9.7.2. Please indicate the outcomes being compared

9.7.3. Statistical test used for the comparison

9.7.4. Result of the test

9.7.5. P value / Confidence intervals

9.8. Comparison IV

9.8.1. Please indicate the intervention groups being compared

9.8.2. Please indicate the outcomes being compared

9.8.3. Statistical test used for the comparison

9.8.4. Result of the test

9.8.5. P value / Confidence intervals

9.9. Comparison V

9.9.1. Please indicate the intervention groups being compared

9.9.2. Please indicate the outcomes being compared

9.9.3. Statistical test used for the comparison

9.9.4. Result of the test

9.9.5. P value / Confidence intervals

9.9. Comparison V

9.9.1. Please indicate the intervention groups being compared

9.9.2. Please indicate the outcomes being compared

9.9.3. Statistical test used for the comparison

9.9.4. Result of the test

9.9.5. P value / Confidence intervals

9.9. Comparison V

9.9.1. Please indicate the intervention groups being compared

9.9.2. Please indicate the outcomes being compared

9.9.3. Statistical test used for the comparison

9.9.4. Result of the test

9.9.5. P value / Confidence intervals

For each comparison conducted in the study, please copy and paste the cells as appropriate

10.1. Organisational setting

10.2. Technological infrastructure

10.3. Instructional Systems Design and Curriculum development

10.4. Delivery

10.5. Advantages of eLearning - as reported by the study authors

10.6. Disadvantages of eLearning - as reported by the study authors

11.1. Source of financing - as reported by the study authors

11.2. Did the intervention undergo a formal accreditation process within the host institution?

11.3. If yes, please describe

11.4. Was the eLearning intervention developed for this study consequently adopted as a formal method for the delivery of education at the host institution?

11.5. If yes, please specify

12.1. Study conclusions - as stated by the study authors

12.2. Limitations of the study - as reported by the study authors

12.3. Was contact with the study authors sought? - If No, please go to section 12.5

12.4. Please indicate the nature of the information requested from the study authors

12.5. Please indicate the results of the request for information

12.6. Additional notes

3: Results of electronic searches

Number of citations yielded by the electronic searches for each bibliographic database

Database	Results	
	Before de-duplication	After de-duplication
MEDLINE	941	806
EMBASE	3206	3123
PsycINFO	334	334
Web of Knowledge	6993	4099
ERIC	146	146
CENTRAL	588	584
Total	12208	9092

4: Characteristics of included studies for online computer-based eLearning

4: Characteristics of included studies for online eLearning

STUDY ID	Methods: STUDY DESIGN	Methods: LOCATION	Methods: COMPARISON	Participants: TOTAL NUMBER	Participants: YEAR OF STUDY	Participants: HEALTHCARE SPECIALITY	Interventions: CHARACTERISTICS	Interventions: TIME AND TECHNOLOGY	Interventions: TEST (OUTCOMES)
[illegible]	RCT	UK	eLearning vs traditional learning	[illegible]	First year	Nursing	CG: students in the control group did not have access to Authentic World but received all the usual support from academic teaching staff<br>IG: students had access to Authentic World, computer software designed to improve medication dosage calculation problem-solving skills	• 7 months<br>• Computer, Authentic World software	Knowledge: general numeracy test
[illegible]	RCT	UK	eLearning vs traditional learning	[illegible]	First year	Nursing	CG: students in the control group did not have access to Authentic World receive all usual support from academic teaching staff<br>IG: Students had access to Authentic World, computer software designed to improve medication dosage calculation problem-solving skills	• 6 months<br>• Computer, Authentic World software	Knowledge: general numeracy test
[illegible]	RCT	Spain	eLearning vs traditional learning	[illegible]	Second year	Physiotherapy	CG: books and notes<br>IG: students had access to a website with detailed sections, including videos, images and self-assessment features	• [illegible] weeks<br>• computer, website	Knowledge: MCQ<br>Skills: OSCE<br>Satisfaction: scoring Likert-scaled questionnaire
[illegible]	RCT	USA	eLearning vs traditional learning	[illegible]	Fourth year	Medicine	CG: students received no [illegible]<br>IG: students used the [illegible] CyberSchool	• [illegible] weeks<br>• computer, website	Knowledge: standardised written examination<br>Satisfaction: survey
[illegible]	RCT	Belgium	eLearning vs traditional learning	[illegible]	Final year	Nursing	CG: students received a lecture<br>IG: an eLearning session on pressure ulcers	• 1 hour<br>• computer, website	Knowledge: [illegible] or more independent assessors said on an equal value [illegible] observation or measurement
[illegible]	RCT	UK	eLearning vs traditional learning	[illegible]	First year	Nursing	CG: students received a face-to-face session which replicated the content and structure of the tutorial<br>IG: students in the intervention group worked through the online tutorial which they accessed via a dedicated area on the university's virtual learning environment	• 1 hour<br>• computer, online access	Skills: all search skills were involved a search of the cumulative index to nursing and allied health literature (CINAHL) on a given topic area
[illegible]	RCT	USA	eLearning vs traditional learning	[illegible]	Second year to fourth year	Medicine	CG: students received lectures with no access to the web tutorial<br>IG1: students in the computer group had access to the computer tutorials on the web<br>IG2: students in this group had access to both the lectures and web tutorials	• [illegible] + N/A<br>• computer with Macromedia Flash, Cold Fusion Studio (Macromedia), RealVideo installed, online access	Knowledge: cognitive assessment instrument<br>Attitudes: Likert-type assessment
[illegible]	RCT	Spain	eLearning vs traditional learning	[illegible]	N/A	Physiotherapy	CG: students received study sessions and were allowed to self-study using documents and books<br>IG: students had access to the web-based intervention entitled *Evaluating, Assessing, and Treating Shoulder [illegible]*	• [illegible] weeks<br>• computer, website	Skills: OSCE<br>Attitudes: scoring Likert scale (1 strongly agree, 5 disagree)<br>Satisfaction: scoring Likert scale (1 strongly agree, 5 disagree)

STUDY ID	Methods			Participants			Interventions		
	STUDY DESIGN	LOCATION	COMPARISON	TOTAL NUMBER	YEAR OF STUDY	HEALTHCARE SPECIALITY	CHARACTERISTICS	TIME AND TECHNOLOGY	TEST / OUTCOMES
Chao [illegible]	RCT	Brazil	e-learning vs e-learning	[illegible]	First year	Medicine	CG: students had access to self-assessments on the Internet that were based on the study books<br>IG: students had access to a multimedia website	• 24 hours<br>• computer, website, HTML, active server pages (ASP) a database (SQL 7.0, Microsoft®)	Knowledge: interactive evaluation about metabolism
Chen [illegible]	RCT	USA	e-learning vs traditional learning	[illegible]	N/A	Health-related majors undergraduate students	CG: students received no treatment and proceeded directly into textbook reading and assessment<br>IG I: students received a visual advance organizer<br>IG II: students used the text advance organizer presenting the same content and explanation as the concept map in a text format	• 1 week<br>• computer, a visual concept map software, text advance organizer	Knowledge: two-part test. The first part included multiple-choice and essay questions, two-part quiz and complete a scenario question was administered 2 weeks after treatment
Chen [illegible]	cRCT	Taiwan	e-learning vs traditional learning	[illegible]	Fourth year	Nursing	CG: students received only paper references and no teaching assistance<br>IG: students received online teaching assistance as a computer-based assistant tool with which students could drill and practice items to help their learning through autonomous self-evaluation	• 10 weeks<br>• computer, online access to the e-learning system	Knowledge: midterm and final test
Carter [illegible]	cRCT	USA	e-learning vs traditional learning	[illegible]	Third year	Medicine	CG: students received identical packets of paediatric distributable paper materials with no other instruction<br>IG I: students received a 2-week ... OSCE—that was taught as a method for identifying underserved patients' health issues<br>IG II: students had access to web material including videos and received the OSCE screening tool instructions via the Internet	• 4 weeks<br>• computer, website, videos	Skills: performance examination<br>Attitudes: used a 5-point Likert scale (1 = strongly disagree to 5 = strongly agree)
DeBate [illegible]	cRCT	USA	e-learning vs traditional learning	[illegible]	N/A	Dentistry	CG: students received no additional training beyond what was delivered in their curriculum<br>IG: students received a web-based training program comprised of three interactive intervention components with section overviews and learning objectives. The content material was presented in various interactive formats	• 3 weeks<br>• computer, website	Knowledge, skills and attitudes were all measured using a 5-point Likert-type questionnaire based on the conceptual framework [illegible]
Erickson [illegible]	RCT	USA	e-learning vs traditional learning	[illegible]	Third year	Pharmacy	CG: students received no intervention<br>IG: students received a web-based tutorial	• 1 hour<br>• computer, website, video	Knowledge: open-ended questions describing step-wise process of taking [illegible] medication<br>Skills: mock standardized patient test
Fernandez [illegible]	RCT	Spain	e-learning vs traditional learning	[illegible]	Second year	Nursing	CG: students were taught in an orthodox clinical skills room and given a set of [illegible] additional resources<br>IG: students had access to 'Moodle' – for watching videos, listening to recordings, reading text, looking at photographs and linking to relevant websites and questions	• 10 weeks<br>• computer, online access	Knowledge: [illegible] examination<br>Satisfaction: 5-point Likert scale and open questions
Hendricson [illegible]	RCT	USA	e-learning vs traditional learning	[illegible]	First year	Dentistry	CG: web-based self-instruction and slide/audiotape self-instruction<br>IG: this group studied the mandibular arch using the slide/audiotape and the maxillary arch using the web-based format	• time N/A<br>• slide and audiotape access, computer and online access for the web-based self-instruction	Knowledge: [illegible] 'fill-in-the-blank' questions<br>Satisfaction: preference survey consisted of [illegible] questions with Likert-style responses

STUDY ID	Methods: STUDY DESIGN	Methods: LOCATION	Methods: COMPARISON	Participants: TOTAL NUMBER	Participants: YEAR OF STUDY	Participants: HEALTHCARE SPECIALITY	Interventions: CHARACTERISTICS	Interventions: TIME AND TECHNOLOGY	Interventions: TEST/OUTCOMES
[illegible] 2009	[illegible]RCT	USA	eLearning vs. traditional learning	[illegible]	Fourth year	Pharmacy	CG: students received no intervention on their community placements<br>IG: students in this group were given a website address to access multimedia lessons which they were required to watch to augment their training and standardise their counselling of patients in the use of inhalers and ear and eye drops	• 1 month<br>• computer with PowerPoint® and Internet capabilities installed for digital videos and audio, searchable text, web server, cd, dvd	Knowledge: [illegible] items post-test
[illegible] 2006	RCT	Germany	eLearning vs. traditional learning	[illegible]	Fourth year + 1 year of medical training	Medicine	CG: students received a paper booklet containing identical content to the intervention, but replaced media with multiple screenshots and text-based descriptions<br>IG: students received an online multimedia course about [illegible]	• time N/A<br>• computer, website	Knowledge: [illegible] items MCQ<br>Skills: 10 standardised tasks and open questions targeted toward a procedural understanding of the operation<br>Attitude: questionnaire on Current Motivation<br>Satisfaction: confidence in the Use of Computers questionnaire
[illegible] 2009	RCT	USA	eLearning vs. eLearning	[illegible]	N/A	Nursing	CG: students received a web-based course on cardiac rhythm interpretation<br>IG: this group worked together on case studies, rather than completing them independently. Students in the experimental group also used online chats to enhance their understanding of course concepts	• [illegible] weeks<br>• computer, website	Knowledge: post-test containing [illegible] items multiple choice questions<br>Satisfaction: attitude toward computer-assisted instruction (16 bipolar adjectives, each measured on a 7-point scale, and three factors (i.e. comfort, creativity, function))
[illegible] 2011	RCT	Thailand	eLearning vs. traditional learning	[illegible]	Fourth year	Nursing	CG: students received lectures and practicals on midwifery<br>IG: students had access to a web-based learning unit around the lecture content for midwifery	• [illegible] weeks<br>• computer capable of supporting interactive graphics, animation and online access	Knowledge: [illegible] scenario questions<br>Skills: performance checklist<br>Attitude: 10 items questionnaire, using a 5-point Likert scale ranging from 1 (strongly disagree) to 5 (strongly agree)<br>Satisfaction: clinical stress questionnaire using a 6-point Likert scale was used for each item ranging from 1 (not stressed) to 6 (highly stressed)
[illegible] 2006	RCT	USA	eLearning vs. traditional learning	[illegible]	Third year	Medicine	CG: students from the previous year served as historical controls to address the second research question that compared students receiving the intervention to those who did not<br>IG I: students had access to three web-based standardised patient cases<br>IG II: students worked in groups of three and conducted a total of three SP encounters. Students rotated one of three roles in each encounter: clinician conducting the encounter, observer completing the checklist on history taking and physical examination items, or observer completing the checklist on communication	• 1 hour<br>• computer, website	Skills: checklist<br>Satisfaction: 14 items satisfaction survey with responses on a Likert-type scale ranging from 1 (poor), 2 (fair), 3 (good), 4 (very good), and 5 (excellent)
[illegible]	RCT	USA	eLearning vs. traditional learning	[illegible]	Second year	Medicine	CG: students received a traditional lecture and group work<br>IG: students received a computer assisted instruction tutorial was developed that covers the essentials of skin examination and the terminology used to describe and classify skin lesion morphology and distribution	• [illegible] days<br>• computer, website	Knowledge: [illegible] questions post-test examination

STUDY ID	Methods			Participants			Interventions		
	STUDY DESIGN	LOCATION	COMPARISON	TOTAL NUMBER	YEAR OF STUDY	HEALTHCARE SPECIALITY	CHARACTERISTICS	TIME AND TECHNOLOGY	TEST/OUTCOMES
[illegible]	RCT	Brazil	e-learning vs traditional learning	NR	Fourth year	Nursing	CG: students had to design a manual without the use of the internet<br>IG: these students had to design a nursing schedule via the website	• time N/A<br>• computer, website	Knowledge: MCQ<br>Satisfaction: research form (interest in the topic)
[illegible]	Canada		e-learning vs traditional learning	[illegible]	Second year	Medicine	CG: students were presented with the real-world [illegible] on pediatric stridor<br>IG: this group was given an online computer-assisted module that covered pediatric stridor	• [illegible] minutes on average<br>• computer with online access with WebCT Vista	Knowledge: MCQ and time to complete the online material<br>Satisfaction: questionnaire
[illegible]	RCT	USA	e-learning vs e-learning	[illegible]	Third year	Medicine	CG: students received web-based training covering benign prostatic hyperplasia and erectile dysfunction topics<br>IG: students received interactive spaced education covering topics benign prostatic hyperplasia and erectile dysfunction	• CG: [illegible] weeks<br>IG: [illegible] weeks<br>• computer with email client and online access	Knowledge: a [illegible]-item adaptive-spaced test and [illegible]-item MCQ
[illegible]	RCT	USA	e-learning vs e-learning	[illegible]	Third year	Medicine	CG: students received [illegible] daily<br>IG: students received daily emails including an evaluative and educational components whereby answers to questions were submitted online	• [illegible] weeks<br>• computer with email client, online access for "MyCourses" course management system	Knowledge: orthodontic examination form for each patient
[illegible]	RCT	USA	e-learning vs traditional learning	[illegible]	Second year	Dentistry	CG: students received only hard copy of dental records<br>IG: this group used digital records on computers	• time N/A<br>• computer, online access	Knowledge: orthodontic examination form for each patient<br>Satisfaction: a survey done at the end of the course for acceptance of the web-based examination
[illegible]	RCT	China	e-learning vs traditional learning	[illegible]	Fourth year	Medicine	CG: students did not receive the workshop, no [illegible] related material during their paediatric rotation<br>IG: students participated in a workshop on clinical reasoning that was web-based	• [illegible] minute lecture, [illegible] minute workshop<br>• computer, online access	Knowledge: clinical reasoning problems (CRP) score and diagnostic thinking inventory (DTI) score<br>Satisfaction: [illegible]-item written questionnaire, each item, students rated their agreement using a Likert scale ([illegible] strongly disagree, [illegible] strongly agree)
[illegible]	RCT	USA	e-learning vs traditional learning	[illegible]	N/A	Medicine	CG: students had to prepare a written exercise on unrelated topics<br>IG I: students completed cases on the computer<br>IG II: two computer cases on low back pain, kidney stones and pneumonia	• time N/A<br>• computer, online access	Knowledge: a [illegible]-question [illegible] final exam that was given throughout the three-year study period. Sixteen questions on pneumonia, low back pain, and kidney stones (CC, computer-case related) were included in the exam<br>Skill: time to complete the exercise<br>Satisfaction: anonymous questionnaire – agreement on a seven-point scale
[illegible]	RCT	Canada	e-learning vs traditional learning	[illegible]	Second year	Medicine	CG: students received a PowerPoint teaching session and were allowed to use two neuroanatomy textbooks<br>IG: students used an access to localization tool exploring cranial nerve lesions	• [illegible] minutes<br>• computer, online access	Knowledge: a [illegible]-question [illegible] final exam<br>Satisfaction: questionnaire and agreement on a seven-point scale
[illegible]	RCT	USA	e-learning vs traditional learning	[illegible]	Second year	Medicine	CG: students were not assigned a password and could not participate in the web discussions<br>IG: students had access to an internet component and used the application to discuss a series of four cases involving substantial ethical problems	• time N/A<br>• computer, online access	Knowledge: final written case analyses<br>Satisfaction: subjective evaluations of the course by both students (using a course-evaluation instrument) and faculty (using an evaluation instrument developed for this study)

STUDY ID	Methods			Participants			Interventions		
	STUDY DESIGN	LOCATION	COMPARISON	TOTAL NUMBER	YEAR OF STUDY	HEALTHCARE SPECIALITY	CHARACTERISTICS	TIME AND TECHNOLOGY	TEST/OUTCOMES
Lu 2009	qRCT	Taiwan	eLearning vs. traditional learning	[illegible]	Second year	Nursing	CG: students only received classroom lectures and skill demonstration<br>IG: students were able to use a web-based course and were able to view the content on demand with access to a chatroom, bulletin board, and email	• time N/R<br>• computer, online access and intervention software	Knowledge: Intramuscular Injection knowledge assessment scale, [illegible] questions<br>Skills: Intramuscular injection skill performance scale, with possible scores ranging from 0 to 100
Maag 2004	RCT	USA	eLearning vs. traditional learning	[illegible]	N/A	Nursing	CG: students independently read and learnt from paper-based mathematical modules<br>IG I: students independently read and learnt from paper-based mathematical modules with images included<br>IG II: students viewed the modules in a multimedia format on a computer screen in the form of a web page	• 1 hour<br>• computer, online access	Knowledge: [illegible] MCQ<br>Attitudes: [illegible] items, statements asking the respondents to specify their level of confidence on a [illegible]-point scale ranging from no confidence at all to complete confidence<br>Satisfaction: student satisfaction survey; the participants were asked to rate aspects of the learning modules on a scale of 1 to [illegible] (1 indicating strongly disagree and [illegible] indicating strongly agree)
Mahnken 2011	RCT	Germany	eLearning vs. traditional learning	[illegible]	Fourth year	Medicine	CG: students looked at imaging procedures and reporting in radiography and radiology without access to the e-learning unit<br>IG: students looked at imaging procedures and reporting in radiography and radiology with access to the e-learning unit and e-case	• time N/R<br>• computer, online access	Knowledge: test comprised of [illegible] items, which were randomly chosen from a pool of [illegible] questions
[illegible] 2013	qRCT	UK	eLearning vs. eLearning	[illegible]	N/A	Medicine	CG: students were given access to a dummy learning package incorporating the outcome measures with no formal learning content<br>IG: students used an online acute breathing difficulty learning package	• [illegible] weeks<br>• computer, online access	Knowledge: post intervention MCQ assessment<br>Attitudes: questionnaire
Mattheos 2004	RCT	Sweden	eLearning vs. eLearning	[illegible]	Second year	Dentistry	CG: students only had access to the case and reference material were available through a static web page<br>IG: students had access to cases and reference material were available through interactive software	• [illegible] weeks<br>• computer containing the intervention software and online access	Knowledge: written essay, written comparison between their answer and that of the expert and oral performance<br>Satisfaction: questionnaire
Meckfessel 2011	RCT	Germany	eLearning vs. traditional learning	[illegible]	Third year	Dentistry	CG: students received didactic lectures and a PowerPoint™ presentation<br>IG: students had access to online modules	• [illegible] weeks<br>• computer with online platform and email client	Knowledge: MCQ<br>Attitudes: questionnaire with answers chosen between 1 and [illegible] (1 = totally disagree, [illegible] = totally agree); attendance of lectures<br>Satisfaction: questionnaire
Meckfessel 2011	RCT	Germany	eLearning vs. traditional learning	[illegible]	Third year	Dentistry	CG: students received a traditional lecture<br>IG: Students had access to traditional face-to-face lectures, but also received emails containing multiple choice questions on the learning content of the lectures	• [illegible] minute sessions<br>• computer with online platform and email client	Satisfaction: questionnaire

STUDY ID	Methods: STUDY DESIGN	Methods: LOCATION	Methods: COMPARISON	Participants: TOTAL NUMBER	Participants: YEAR OF STUDY	Participants: HEALTHCARE SPECIALITY	Interventions: CHARACTERISTICS	Interventions: TIME AND TECHNOLOGY	Interventions: TEST/OUTCOMES
[illegible] 2008	RCT	USA	e-learning vs traditional learning	[illegible]	Third year	Medicine	CG: students received traditional written material<br>IG: students received a web-based interactive program created to teach seizure disorders, including the basic teaching material using an interactive format	• 2 days<br>• computer, online resources with audio and video support	Knowledge: 10 MCQ<br>Attitudes: survey using a Likert scale to assess the students' attitude toward learning, motivation, and feedback perception
[illegible] 2008	RCT	Australia	e-learning vs traditional learning	[illegible]	Fourth year	Medicine	CG: students received standard lecture material without any additional learning materials other than those recommended to all students<br>IG1: students received same case studies as control, but in an interactive computer-based format and questions answered with detailed feedback<br>IG2: students received both the standard clinical material plus interactive computer-based case studies	• [illegible] weeks<br>• computer with Media software installed, online access and CD drive	Knowledge: [illegible] MCQ and [illegible] modified question case<br>Satisfaction: a questionnaire of their perceptions of the value of these resources
[illegible] 2008	RCT	Germany	e-learning vs traditional learning	[illegible]	Second and third clinical semester	Dentistry	CG: students received a lecture presenting the same content, including the same photos, with the use of PowerPoint®. It was possible for the students to question or discuss during and after the lecture<br>IG: students received a module including [illegible] cases of clinical situations, slides and graphics. It was linked with a text in the virtual module for self-assessment, with case presentations, and further resources	• self-study as they wanted (average [illegible] minutes)<br>• computer, online resources	Knowledge: [illegible] MCQ and three [illegible] questions<br>Satisfaction: [illegible] statements about educational and enjoyment values of the teaching methods were given and the students were asked to express their agreement on a [illegible]-point Likert scale ([illegible]= no agreement, [illegible]= full agreement)
[illegible] 2009	RCT	USA and Brazil	e-learning vs traditional learning	[illegible]	Second and third year	Medicine, nursing, physiotherapy	CG: students received a writing workshop for biomedical sciences module offered with face-to-face instruction<br>IG: students used a writing workshop conducted in a virtual environment using virtual tools (VoIP, emails) and templates provided as tools	• time N/A<br>• computer with Voice over the PowerPoint®, Internet Protocol (VoIP: SKYPE) installed, [illegible] access and email support	Knowledge: scored using a [illegible]-subgroup quality scale<br>Satisfaction: Likert scale, with responses ranging from strongly disagree to strongly agree
Raupach 2009	RCT	Germany	e-learning vs traditional learning	[illegible]	Fourth year	Medicine	CG: students received traditional classroom teaching in group study on case histories<br>IG: students took web-based collaborative teaching module on the differential diagnosis of dyspnea	• [illegible] weeks<br>• web-based collaborative teaching module. [illegible] learning management systems (CLIX) facilitating live chat, asynchronous group discussions and the exchange of documents was used to create the online module	Knowledge: [illegible] MCQ (pretest) problem-solving questions (final test)<br>Satisfaction: questionnaire
Raupach [illegible]	RCT	Germany	e-learning vs traditional learning	[illegible]	Fourth year	Medicine	CG: no description given<br>IG: students received access to an online module consisting in addition to the traditionally taught module	• [illegible] hours accessed per week over [illegible] weeks<br>• computer with online access for the web-based learning management system (CLIX)	Knowledge: [illegible] MCQ<br>Satisfaction: survey conducted at the start and at the end of the [illegible] week course

STUDY ID	Methods			Participants			Interventions		
	STUDY DESIGN	LOCATION	COMPARISON	TOTAL NUMBER	YEAR OF STUDY	HEALTHCARE SPECIALITY	CHARACTERISTICS	TIME AND TECHNOLOGY	TEST/OUTCOMES
[illegible]	RCT	Canada	e-learning vs. traditional learning	[illegible]	Third and fourth year	Medicine	CG: students received no intervention and were tested only<br>IG: students completed multimedia-based computerized procedure tutorials	• [illegible] minutes<br>• computer; online access with support for digital images, short video clips, and instructional text, Internet, hospital website	Knowledge: 10-item multiple-choice examination
[illegible]	RCT	Finland	e-learning vs. e-learning	[illegible]	Third year	Medicine	CG: students received course material offered through conventional technology and were able to engage in an email discussion with an instructor for a noninteractive web-based learning<br>IG: students used WebCT group access to the study materials, discussion forums, resource agents	• 3 weeks<br>• computer; online access of WebCT	Knowledge: [illegible]-item multiple choice, matching and short answer questions<br>Satisfaction: 'Impact of ICT' consisted of [illegible] items, and the 'Learning Experience' consisted of 6 items on a 4-point Likert scale
[illegible]	RCT	USA	e-learning vs. e-learning	[illegible]	Second to fourth year (mainly third year)	Medicine	CG: students received a PowerPoint™ presentation<br>IG: students received an online, self-paced, sleep medicine learning module provided sleep education	• 1 month<br>• computer; PowerPoint™ installed and online access	Knowledge: measured with a modified version of the Dartmouth Sleep Knowledge and Attitude survey
[illegible]	RCT	Sweden	e-learning vs. e-learning	[illegible]	First year	Dentistry	CG: students were able to watch a sequential web-based video<br>IG: students had access to the same web-based video but split into 6 different parts	• 1 week<br>• computer; online access	Knowledge: written test with 6 questions<br>Skills: performance was rated on a scale from 1 to 4 (poor—excellent) for each of 4 different stages of hand washing<br>Attitude: a questionnaire with 10 questions regarding students' attitudes towards video-based instruction and learning was filled in after completion of all the phases. 7 of these questions were answered on a Visual Analogue Scale (VAS, 0—100 mm) and the remaining 3 were open text questions
[illegible]	RCT	The Netherlands	e-learning vs. traditional learning	[illegible]	Second year	Medicine	CG: students received photocopies of pages of an occupational medicine workbook, practice guideline material and scientific article<br>IG: students elaborated 3 occupational medicine e-learning cases on individual computers	• half a day<br>• computer; online access	Knowledge: 20 questions, divided in multiple-choice, open-ended and true/false questions<br>Attitude: 12 questions on a 4-point Likert scale was used with anchors ranging from disagree to agree<br>Satisfaction: 4 questions: 1 was a general rating for satisfaction (range 1–10), 2 questions about satisfaction with the course content (4-point Likert scale, minimum score 4, maximum 8) and 3 questions about satisfaction with learning (4-point Likert scale, minimum score 3, maximum [illegible])
[illegible]	RCT	USA	e-learning vs. traditional learning	[illegible]	Fourth year	Medicine	CG: students received a live lecture and then group discussion work<br>IG: students received an Internet-based PowerPoint™ slide presentation from the same lecture with the option of audio	• time N/A<br>• computer; PowerPoint™ installed and online access and audio	Knowledge: an open book exam that has been used in the course for 1 year<br>Satisfaction: students rated their satisfaction with their lecture on a 5-point Likert scale and they provided narrative about their experiences with the live lecture and the online lecture

STUDY ID	Methods			Participants			Interventions		
	STUDY DESIGN	LOCATION	COMPARISON	TOTAL NUMBER	YEAR OF STUDY	HEALTHCARE SPECIALITY	CHARACTERISTICS	TIME AND TECHNOLOGY	TEST/OUTCOMES
[illegible]	RCT	USA	eLearning vs eLearning	[illegible]	Third and fourth year	Medicine	CG: students received an online lecture consisted of an Internet-based PowerPoint™ slide presentation with no audio narration<br>IG: students received an online lecture consisted of an Internet-based PowerPoint™ slide presentation with audio narration	• time N/S<br>• technology N/S	Knowledge: post-test<br>Included discussion questions that solicited approaches to a hypothetical patient<br>Satisfaction: students rated their satisfaction with their lecture on a 5-point Likert scale and they provided narrative about their experiences
[illegible]	RCT	USA	eLearning vs traditional learning	[illegible]	Third year	Medicine	CG: students received lectures on general surgery and surgical core skills<br>IG: students received lectures on general surgery and surgical core skills via videoconference	• 6 hours<br>• technology N/S	Knowledge: quiz questions
[illegible]	RCT	Australia	eLearning vs traditional learning	[illegible]	N/A	Medicine	CG: students were taught on the standard neonatology teaching programme<br>IG: students had access to the standard neonatal educational programme and additional unlimited online access to the multimedia [illegible] Baby Check Learning Module	• [illegible] hours<br>• technology N/S	Skills: formative assessment of newborn examination using a standardised checklist<br>Satisfaction: measured using a Likert-like questionnaire
[illegible]	RCT	Switzerland	eLearning vs traditional learning	[illegible]	Third year	Medicine	CG: students received normal lecture including video demonstration and access to a self-directed web-based learning module on smoking cessation<br>IG I: students received a self-directed web-based learning module on smoking cessation<br>IG II: students' patient-centred counselling through role-play in small groups: one medical student acted as a smoker and another one acted as the physician, following standardised case description<br>IG III: students supervised interaction with real patients; counselling encounters with real patients who smoke	• [illegible] hours<br>• computer, online access	Knowledge: [illegible] MCQ<br>Skills: [illegible] OSCE
[illegible]	RCT	USA	eLearning vs traditional learning	[illegible]	Third year	Medicine	CG: students received two PowerPoint™ presentations<br>IG: students had access to web-based learning course	• 1 hour<br>• computer, online access and software for the web-based [illegible] Interactive Medical Software	Knowledge: [illegible]-question post-intervention test, long-term retention was tested using [illegible]-question test [illegible] days after the first test
[illegible]	RCT	Australia	eLearning vs traditional learning	[illegible]	N/A	Medicine	CG: students received traditional methods of teaching and no access to the intervention<br>IG: students in this group not only underwent the traditional teaching, but also were given access to a Virtual Ophthalmology Clinic	• time N/S<br>• computer, online access	For knowledge and satisfaction: a [illegible]-item multiple-choice ophthalmic knowledge-based pre- and post-test and student satisfaction questionnaire was administered
[illegible]	cRCT	Australia	eLearning vs traditional learning	[illegible]	Second year	Pharmacy	CG: students worked in small group workshops<br>IG: students used an Internet-based tutorial for which they could review any content any time as they wished	• [illegible]<br>• computer and online access	Skills: post-test assessing their inhaler technique against a checklist

STUDY ID	Methods			Participants			Interventions		
	STUDY DESIGN	LOCATION	COMPARISON	TOTAL NUMBER	YEAR OF STUDY	HEALTHCARE SPECIALITY	CHARACTERISTICS	TIME AND TECHNOLOGY	TEST /OUTCOMES
Truncali 2011	RCT	USA	elearning vs. traditional learning	94	First year	Medicine	CG: students received a PowerPoint® presentation and video<br>IG: students received information through interactive multimedia slides, with external resources and 2 video cases with questions	• 3 days before lecture until 1 day before OSCE<br>• computer (Java enabled) and online access	Knowledge: 12 item multiple-choice test<br>Skills: OSCE<br>Attitude: a 12-item attitudes questionnaire with another 4-point scale (1 = strongly disagree, 2 = somewhat disagree, 3 = somewhat agree, 4 = strongly agree) was used to assess students' feeling toward patients with substance abuse and their treatment
Wang 2009	RCT	China	elearning vs. traditional learning	123	Second year	Nursing	CG: students recevied a slideshow presentation<br>IG: students received an online based resource in which had to self learn the topic which also allowed online discussion	• time N/A<br>• computer and online access	Knowledge: MCQ<br>Satisfaction: questionnaire
Yeung 2012	RCT	UK	elearning vs. traditional learning	78	Second year	Medicine	CG: students received a text/image based document<br>IG: students received an online module with different views and an unlimited time allocation	• time N/A<br>• computer and online access	Knowledge: MCQ<br>Satisfaction: subjective questionnaire using a 5-point Likert scale

CG: Control group

IG: Intervention group

*Publication contained two studies

5: Risk of bias in included studies

In this section we will cover risk of bias for the 52 included parallel RCTs studies. Risk of bias for the eight included cluster RCTs will be presented separately in a consecutive section.

Thirty-one of the studies were considered to be of low quality because of high risk of bias. [1-30] Twenty-nine of the studies [31-46] had one or more categories classified as an unclear risk of bias, especially regarding the allocation of participants to intervention groups. There was only one study [47] with all the categories classified as low risk of bias - see Figure 2 (Risk of bias graph) and Figure 3 (Risk of bias for each individual parallel RCT separately).

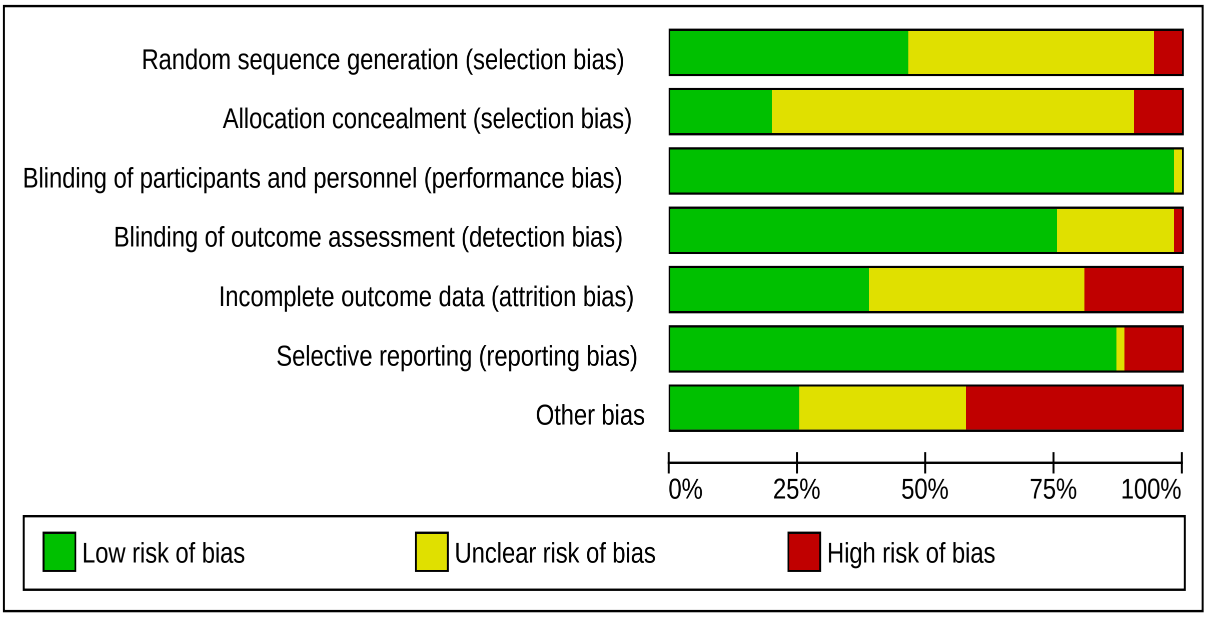

Figure 2. Risk of bias graph

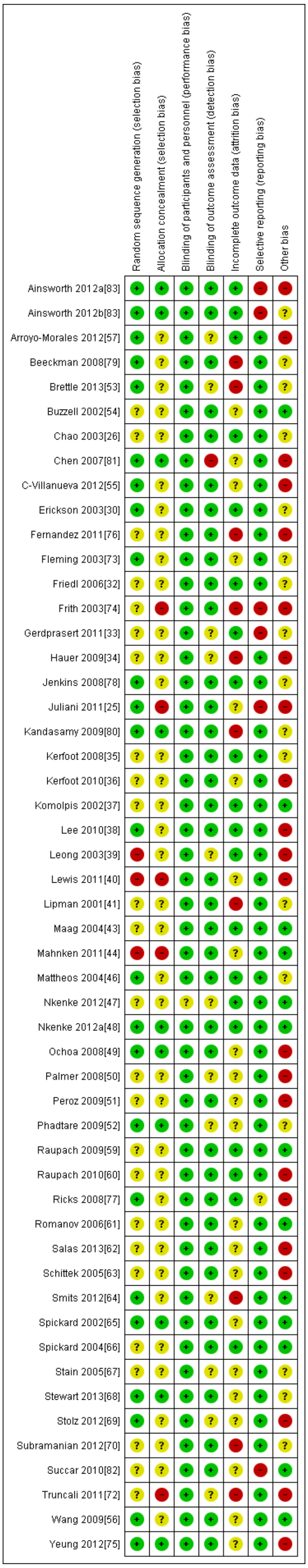

Figure 3. Risk of bias for each individual parallel RCT separately

Random sequence generation and allocation (selection bias)

Most studies (25 of the 52 studies, 48%) included little or no information about the *random sequence generation* and were therefore classified as having an unclear risk of bias. [1, 3, 8, 11, 12, 14, 18, 19, 22-24, 30, 31, 33, 35, 38, 40, 41, 43, 44, 47-50] Only three of the 52 studies (6%) had a high risk of bias for *random sequence generation*. One [7] of these studies generated the allocation sequence by assigning students to an odd or even number in the order with which they were entering the room. The other two studies violated the randomization plan by letting students choose between three alternative assignments freely [6] or by assigning students to the study groups in a consecutive way, i.e. based on the order in which they undertook a specific internship.[9] The Random sequence generation was classified as judged to result in a low risk of bias for 24 [2, 5, 10, 13-16, 20, 21, 25, 27-30, 32, 36, 37, 39, 42, 45, 46, 51, 52] of the 52 studies (46%).

The method used in the majority of the cases to generate a random number sequence was computer software [5, 13-16, 21, 25, 27-30, 37, 39, 46, 51, 52]. Furthermore, one study used name drawing, [2] two studies used a random number table [20, 45] and one other used “odd” and “even” conditions from a random number series.[39]

There was no information about the allocation concealment method in 37 [1, 3, 4, 6, 8, 11-14, 16-22, 26-28, 31-36, 38-42, 44-46, 50, 53] out of the 52 trials (73%) and therefore these studies were classified as having an unclear risk of allocation bias. Five studies (10%) had high risk of allocation bias. One of these studies [2] facilitated its randomization process by drawing the name inside the class room within the presence of all class members; and another study [23] posted their randomization result on the website four days before the lecture. The other three studies [7, 9, 24] reported having problems in their randomization procedures, which made the allocation concealment impossible. Ten studies (19%) had a low risk of bias for allocation concealment. Four [25, 29, 30, 52] of these studies generated the random numbers on a computer and delivered them in a way that ensured concealment of allocation. Two studies from the same paper used the centralized randomization process.[15] The remaining four studies [10, 15, 36, 37, 42] used opaque envelopes for concealment.

Blinding (performance bias and detection bias)

The risk of bias assessment for blinding of participants and personnel focused only on the knowledge and skills outcomes. The risk of bias was classified as low for all but one study,[47] even though blinding of participants and personnel was not possible in any of these studies because of the nature of the intervention. The only study considered to have a high risk of bias related to blinding of participants and personnel [47] did not report any outcome on knowledge or skill. Our overall assessment for the performance bias was based on the fact that only the 43 studies investigating knowledge [2, 3, 5-12, 15-17, 20-23, 25-30, 32, 33, 35-40, 42, 44-46, 50, 53-58] and the 11 studies [1, 3, 6, 13, 14, 16, 21, 23, 32, 33, 52] measuring skills had an objective outcome assessment. Therefore, the assessment was considered impervious to the student’s opinion about the teaching method. As indicated before, subjective outcomes such as attitudes and student satisfaction were not included in the risk of bias assessment for *blinding of participants and personnel*. These outcomes are more prone to performance bias when participants aren’t blinded due to their subjective nature and also focusing on attitudes and student satisfaction would therefore have resulted in a high risk of bias in all studies.

Thirty-nine of the 52 included parallel RCTs (75%) were considered to be at low risk of bias for the *blinding of outcome assessment*. The risk of bias was considered low risk not only in studies where all outcome assessors were blinded [8, 14, 15, 19, 32, 42, 43, 50, 52] but also in studies with unblinded assessors as long as the method of outcome assessment included no element of interpretation and a classification of a result could be done unambiguously [2, 4, 5, 7, 9, 10, 12, 17, 18, 22, 24-29, 31, 33-36, 38-41, 45, 46, 53], e.g. the only assessment was a multiple choice test. Twelve studies (22%) were rated as having an unclear risk of bias due to the lack of information about blinding of the outcome assessors [1, 3, 6, 11, 13, 16, 20, 21, 23, 36, 37, 44]. Only one study [30] had high risk for detection bias because it reported a mixed knowledge outcome for which a part of the result was considered unblinded.

Incomplete outcome data (attrition bias)

As a consequence of the fact that none of the students were blinded there is a high risk of attrition bias for any outcome that relies on active participation of students for follow-up (e.g. answering a questionnaire on attitudes and satisfaction and taking a knowledge test).

A substantial number (10 out of 42, 19%) of the studies did not report complete outcome data (e.g. only reported the mean test score but did not report the number of students who were analysed) or had differential drop-out rates in the different intervention groups and were classified as high risk of bias. Two of high risk of bias studies (4%) showed a difference in the attrition/exclusion rates between the experimental groups. [1, 29] Five studies (12%) that were classified as having a high risk of bias had missing/unreported data and did not account for or comment on that. [13, 20, 23, 24, 28] The remaining three studies reported inconsistent sample sizes.[8, 22, 26] Twenty (38%) studies were classified as having a low risk of bias for incomplete outcome data. [3, 5, 6, 15-17, 27, 31-36, 40, 43, 46, 47, 50, 51] These studies reported whether attrition and exclusion had occurred. The information provided regarding the reason for not analysing all participants was either similar for the groups being compared and/or showed only a small and statistically insignificant difference between the studies.

Because details of attrition and exclusion were not reported, 22 studies (42%) were classified as having an unclear risk of bias for *incomplete outcome data* [2, 4, 7, 9-12, 14, 18, 19, 21, 25, 28, 38, 39, 41, 42, 44, 45, 52, 53]In these studies it was not clear if there was any level of attrition among the experimental groups at all.

Selective reporting (reporting bias)

The majority of studies (45 out of 52,87%) were rated as having a low risk of selective reporting bias [1, 4-14, 16-23, 25, 26, 28-47, 50-52]. The assessment of selective reporting bias required the authors to report results for all outcomes mentioned in the methods sections of the published articles; protocols were not available to our reviewers. Only one study (54) (5%) was rated as having unclear risk of selective reporting bias because the authors presented more results than the outcomes mentioned in the method section. Six out of the 52 studies (12%) were rated as having a high risk of selective reporting bias. Four of these studies [2, 3, 24, 53] did not report the results in full, making it impossible to get separate results for each group. Two studies presented in the same article extended their study period to have a long-term outcome.[15]

Other potential sources of bias

Volunteer bias is an important and sometimes almost inevitable problem in studies assessing different ways of learning. Therefore, volunteer bias resulted in a high risk of bias classification in 16 of the 52 included studies (31%) [5-7, 10, 12, 14-17, 19, 23-27, 30] It was unclear whether volunteer bias was a problem in 15 of the 52 studies (29%) and therefore they were classified as having an unclear risk of bias. [3, 13, 21, 22, 28, 29, 31-33, 37, 44-46, 51, 52] Among them, nine of the studies did not provide information for the recruitment process [3, 21, 22, 31-33, 44, 45, 51], while six studies [13, 28, 29, 37, 46, 52] approached all the students but not all of them agreed to participate in the trial. Twenty studies (39%) recruited or approached entire class rooms or the entire year and were therefore at low risk of volunteer bias. [1, 2, 4, 8, 9, 11, 18, 20, 34-36, 38-43, 47, 50, 53]

We classified six more studies (12%) as having a high risk of other potential sources of bias. [1, 2, 4, 11, 13, 18, 21] Two studies suffered from imbalanced experimental groups where more material or information was given in one group compared to the other. In one study the web-based intervention group was not exposed to comparable knowledge/skills [21] as was the control group. In another study [18] the experimental groups were not provided with equivalent academic education because the students in the control group were provided only with facts that were taken from a website that is accessible to the general population. Contamination (i.e. the control group was also exposed to the eLearning intervention) was also a concern in one study (6%), which was categorised as having a high risk of bias.[11] However, it is possible that contamination occurred in several of the other included trials as it is likely that students shared material with course mates who were randomised to a different group. Three studies were rated as having a high risk of other bias because one study used a historical control group,[1] one allowed some of the students to hand in their assessment, a schedule, in person rather than electronically,[2] while another study reported that authors had a conflict of interest with spaced education.[4]

We classified *other bias* as high risk of bias if one of the elements assessed was of high risk even when other elements were rated as having an unclear or low risk of bias. For example, if there was a high risk of volunteer bias but an unclear risk for using comparable learning interventions between experimental groups we would classify it as having a high risk of bias. Please refer to Figure 3 for the assessment per study.

Risk of bias in cluster RCTs

Eight studies included in our review were cluster RCTs. [54-61] In these studies one or more risk of bias items were categorised as high risk of bias. Therefore, the methods and analyses employed in these cluster RCTs were generally not judged to be of high quality.

Recruitment bias was not addressed in two [58, 59] of the eight included studies. Two other studies were assessed as high risk for recruitment bias because they applied the randomization process before recruiting the participants. [55, 56] The remaining studies [54, 57, 60, 61] that were judged to be of low risk of recruitment bias had provided sufficient information on the participant flow and randomization process.

Baseline characteristics differed between the intervention and control group in two studies. [60, 61] In three other studies there was a difference in educational level, primary care clerkships or academic grades for the previous semester at baseline. These imbalances were judged to be of high risk of affecting the outcome [55, 58] or confirmed to have modified the effect.[59] Three studies [54, 56, 57] provided no information on baseline characteristics and whether these were different between the groups.

None of the studies reported loss of entire clusters. However, three studies [55, 57, 61] reported loss of individual participants and three additional studies had a high [60] or imbalanced [58] drop-out rate or reported inconsistent numbers,[54] all of which resulted in a high-risk of bias classification. One study reported attrition in both groups but was judged as having an unclear of risk of loss of clusters for providing no further information.[59] One study [56] reported attrition but was judged to be of low risk of bias as the attrition was limited and could not have affected the results.

The data analysis of two studies [56, 60] accounted for the cluster unit. The rest of the cluster RCT studies [14, 54, 55, 57, 61, 62] suffered from unit of analysis error (i.e. incorrectly analysed participants as independent individuals rather than the unit they were randomized in). Therefore there is a high risk of false positive conclusions in these studies.

It was unclear whether or not volunteer bias had occurred in two studies. [54, 58] The remaining six studies [55-57, 59] were all categorised as having a low risk of volunteer bias.
